# Advances in Biotechnological GABA Production: Exploring Microbial Diversity, Novel Food Substrates, and Emerging Market Opportunities

**DOI:** 10.3390/ijms27010306

**Published:** 2025-12-27

**Authors:** Fabian Hernandez-Tenorio, Mateo Mejía-Rúa, Luz Deisy Marín-Palacio, Bernadette Klotz-Ceberio, David Orrego, Catalina Giraldo-Estrada

**Affiliations:** 1 Escuela de Ciencias Aplicadas e Ingeniería, Universidad EAFIT, Medellín 050022, Colombia; fehernandt@eafit.edu.co (F.H.-T.); mmejiar14@eafit.edu.co (M.M.-R.); lmarinpa@eafit.edu.co (L.D.M.-P.); 2 Alpina Productos Alimenticios S.A.S. BIC, Sopó 251001, Colombia; bernadette.klotz@alpina.com (B.K.-C.); david.orrego@alpina.com (D.O.)

**Keywords:** lactic acid bacteria, gamma-aminobutyric acid, fermentation, *Lactobacillus*, bibliometric analysis

## Abstract

Gamma-aminobutyric acid (GABA) is a non-protein amino acid distributed in nature by different types of organisms and microorganisms. GABA has been widely studied for its different physiological functions and industrial applications. Its production is mainly carried out through fermentation processes using lactic acid bacteria (LAB), which are of particular interest because they are safe and possess high glutamate decarboxylase enzyme activity. However, GABA production can vary among different LAB species and is affected by culture conditions. Therefore, strain development and selection, as well as optimization of fermentation parameters, are essential to increase GABA yields and meet the needs of industrial demand. This review quantitatively analyzes recent advances in fermentative GABA production, showing a sustained increase in publications and a predominance of chromatography-based quantification methods (approximately 68%), mainly using pre-column derivatization. Optimized fermentation strategies, supported by statistical and artificial intelligence models, have achieved GABA concentrations of up to 90 mM. In parallel, in silico genomic and metabolic analyses revealed the widespread distribution of key GABA biosynthesis and transport genes among LAB, supporting their selection and engineering. Overall, the integration of advanced analytical methods, bioinformatics-guided strain selection, and computational process optimization emerges as a key strategy to enhance GABA productivity and support future industrial-scale applications.

## 1. Introduction

Gamma-aminobutyric acid (GABA) is an organic compound defined as a non-protein amino acid considered an important inhibitory neurotransmitter in the central nervous system of mammals [1]. Its biosynthesis occurs naturally in food products derived from plants and animals. However, these sources contain low levels of GABA; therefore, microbial fermentation is used as an alternative to produce food-grade and pharmaceutical-grade GABA, as it offers the possibility of increasing GABA yield. GABA-producing microorganisms include fungi, bacteria, and yeasts; among these, the group of bacteria known as lactic acid bacteria (LAB) are being investigated as the most promising microorganisms for obtaining significant amounts of GABA, due to the high activity of the enzyme glutamate decarboxylase (GAD), which allows the decarboxylation of L-glutamic acid [2]. LAB are Gram-positive bacteria with coccoid or bacillary morphology, non-spore forming, and acid tolerant [3]. These microorganisms are capable of producing GABA in fermented foods such as kimchi, fermented soybeans, cheese, and yogurt, among others. These advances in microbial GABA production have driven increasing interest in this compound, not only as a functional metabolite in fermented foods but also because of its broad physiological effects and industrial relevance.

Different industries are interested in GABA due to its physiological functions, including anti-obesity, anti-diabetic, and anti-depressant effects. In addition, this amino acid influences DNA and protein synthesis in the brain and helps relieve stress, anxiety, and fatigue by regulating blood pressure and increasing the body’s energy levels. It also improves visual function, memory, and reduces the growth of some tumors, among other effects [4]. Other applications include its use as a precursor for the synthesis of 2-pyrrolidone, a monomer used in the production of polyamide polymers [5]. As a result, GABA has gained high commercial value, and growing health awareness and self-care trends continue to stimulate scientific and technological interest in its production and application [6]. Consistent with this growing interest, the global GABA market is projected to reach approximately USD 143.3 million by 2033, with a compound annual growth rate (CAGR) of about 5% between 2023 and 2033 [7]. This indicates that the demand for GABA products has increased over the past few years. Therefore, there is a growing need to enhance efficient GABA production systems to meet this demand. Among the GABA production strategies, chemical and biological synthesis are the most common, with the latter being considered the most promising option due to high catalytic efficiency, environmental compatibility, and mild reaction conditions [8].

Within the biological framework, the *Lactobacillus* genus comprises numerous GABA-producing species, including *Lactiplantibacillus plantarum*, *Lacticaseibacillus paracasei*, *Lactobacillus helveticus*, *Limosilactobacillus fermentum*, *Lactobacillus delbrueckii* subsp. *Bulgaricus*, *Lactobacillus buchneri*, and *Levilactobacillus brevis*, among others. Other genera capable of producing GABA have also been studied, including *Weissella*, *Propionibacterium*, *Pediococcus*, *Leuconostoc*, and *Enterococcus* [9]. Although some LAB strains have demonstrated a specific ability to produce GABA, challenges related to strain performance, fermentation optimization, and analytical standardization remain insufficiently integrated in the current literature. Consequently, improving fermentation parameters, as well as strain development and selection and the implementation of efficient quantification methods, are highlighted as important strategies for improving GABA production performance [10]. In this context, the present review aims to provide a comprehensive and quantitative assessment of recent advances in fermentative GABA production. In addition to summarizing microbial sources and fermentation strategies, this review incorporates a bibliometric analysis to identify publication trends over the last decade, evaluates in vitro and in silico GABA quantification methodologies, and discusses the application of bioinformatics and statistical modeling in strain selection and fermentation optimization. By integrating analytical, biological, and technological perspectives, this review seeks to identify current limitations and future opportunities for the development of efficient and scalable GABA fermentation processes aligned with industrial and market demands.

## 2. Bibliometric Analysis

GABA is a non-protein amino acid that acts as a depressant neurotransmitter. It has health benefits in cardiovascular diseases, in the reduction in anxiety, in the control of diabetes, among others. Due to its multiple functions, GABA is an important component in the development of various pharmaceuticals and functional foods [11]. GABA production primarily relies on microbial fermentation, with LAB being recognized as one of the most efficient producers due to their capacity to generate higher levels of GABA and their economic viability as starter cultures. However, the ability to produce GABA varies markedly among different LAB strains and different culture conditions [2]. Therefore, in the present review, it was pertinent to analyze trends in fermentative GABA production by implementing scientometric tools. For this purpose, a systematic search was carried out over the last 20 years, based on search terms selected to retrieve information related to GABA-producing strains, factors affecting GABA fermentative production, and current production trends. The information was compiled from the Scopus database using the following equation TITLE-ABS-KEY ((“GABA” AND “gamma-aminobutyric acid”) AND (“LAB” OR “bacteria” OR “lactobacillus”) AND (“production” OR “fermentation”) AND (“culture” OR “pH” OR “temperature” OR “culture medium” OR “fermentation time”)) AND PUBYEAR > 2003 AND PUBYEAR < 2025 AND (LIMIT-TO (DOCTYPE, “ar”) OR LIMIT-TO (DOCTYPE, “re”)). The information collected was purified to avoid repetition of terms. The following software was used: VOSviewer versión 1.6.16 (Leiden University, Leiden, The Netherlands), Bibliometrix (University of Naples Federico II, Naples, Italy) of R commander (×64. 4.1.0), and CorTextManager (INRAE, Noisy-le-Grand, France).

**Figure 1:**
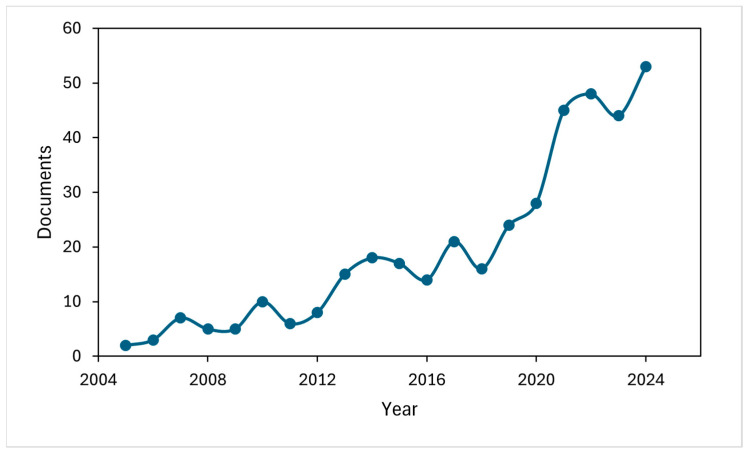
Increasing trend of publications on fermentative GABA production.

Figure 1 illustrates the annual trend of publications on fermentative GABA production, revealing a significant increase of 56.04% in the last five years (2020–2024). This increase probably reflects the growing scientific and industrial interest in the potential benefits and applications of GABA in various fields, such as agriculture, food and pharmaceutical industries. Additionally, it was observed that, in 2024, 16.98% of the total publications exceeded five citations, while in 2023, this figure amounted to 38.63%, suggesting the relevance of studies on fermentative GABA production in current scientific research.

The bibliometric analysis also allowed us to identify the most cited studies on fermentative GABA production. Table 1 presents the document with the highest impact with 925 citations and 66.07 citations per year, corresponding to a research article published by authors affiliated with the Alimentary Pharmabiotic Centre, Biosciences Institute, University College Cork of Cork, Ireland. In this work, the production of γ-aminobutyric acid was investigated in 91 bacteria of intestinal origin. The production capacity of the intestinal strains was evaluated from an anaerobic fermentation based on feces, supplemented with 30 mg/mL monosodium glutamate (MSG) and pH controlled. Four strains of *Bifidobacterium* and one of *Lactobacillus* were identified as producing GABA, with *Levilactobacillus brevis* DPC6108 being the most efficient, with up to 100% conversion of MSG to GABA. The authors highlighted that the identification of the optimal conversion of MSG to GABA from commensal gut microbiota strains and its demonstration in vivo conditions suggest new approaches for modulation of the microbiota to promote health [12]. On the other hand, the review article with the highest number of citations (423) was identified as being used for its significant contributions in GABA production. This article focused on analyzing GABA-producing microorganisms and optimal fermentation conditions. It was suggested that factors such as temperature, medium additives, culture time, and pH are relevant to achieve maximum GABA production [8]. For example, pH is a key factor in GABA synthesis in LAB because it affects GAD activity and the growth of microorganisms. Changes in pH enhance the activation of the GABA pathway and help maintain cellular homeostasis. It has been reported that the optimal pH for effective GABA production ranges from 3.5 to 5.0 and that GAD activity is significantly lost at pH around 7 [9]. Temperature also influences GAD activity, and it varies in different LAB species. S.Y. Yang et al. [13] reported that temperature of 34–37 °C increased GAD activity, while increasing temperature from 37 to 46 °C decreased GAD activity. Another important factor for GABA production is the supplementation of the culture medium with pyridoxal 5′-phosphate (PL) and L-glutamic acid. PLP is a GAD cofactor that promotes increased GAD activity. As for L-glutamic acid, it proves to be essential as its increase stimulates GAB activity and increases GABA production via the GABA shunt metabolic pathway [14].

**Figure 2:**
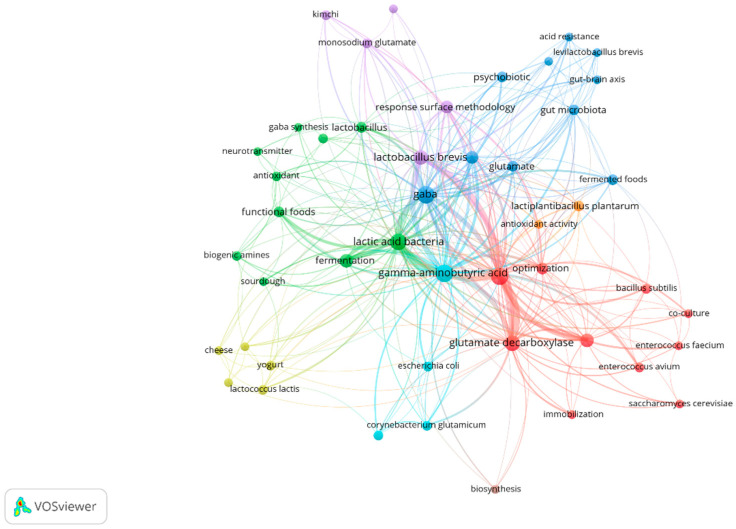
Bibliometric keyword network of authors in publications on fermentative GABA production.

On the other hand, the co-occurrence map allowed us to determine the most frequently cited keywords in research on fermentative GABA production. In Figure 2, the color-coded clusters represent groups of keywords that co-occur frequently in the literature. Strain types identified included *Levilactobacillus brevis*, *Lactiplantibacillus plantarum*, *Lactococcus lactis*, *Corynebacterium glutamicum, Enterococcus avium*, *Bacillus subtilis*, *Enterococcus faecium*, and *Escherichia coli* (red, purple, blue, yellow, orange, and light blue clusters). Additionally, fermented beverages and foods such as cheese, yogurt, and kimchi were used for the isolation of GABA-producing microorganisms (yellow and purple clusters). Furthermore, terms related to GABA production and biosynthesis such as sodium glutamate, immobilization, fermentation, glutamate decarboxylase, optimization, response surface methodology (red, purple, and green cluster) were identified.

**Figure 3:**
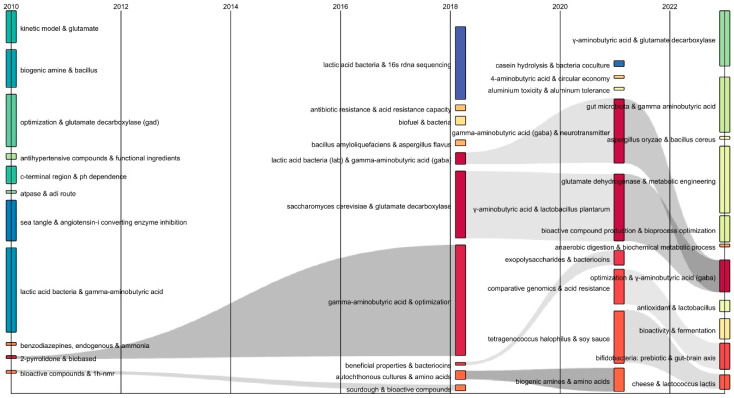
Sankey diagram of author keywords in publications on fermentative GABA production.

Figure 3 shows the Sankey diagram organizes keyword combinations into temporal blocks, where each flow line represents the transition of research topics over time (located from left to right). The width of the lines represents the frequency of co-occurrence of the keyword combinations. The gray flows allow the chronological progression of research themes to be tracked, from early studies (left side) to more recent focuses (right side) [20]. From 2010 through 2018, the keyword combinations “2-pyrrolidone and biobased” and “bioactive compounds and 1 h-nmr” were identified and converged to “gamma-aminobutyric acid and optimization” and “sourdough and bioactive compounds”. This suggests that GABA has also been investigated as a raw material to produce 2-pyrrolidone, a precursor for the synthesis of nylon 4. It is important to note that 2-pyrrolidone is a petrochemical product, so its production generates environmental concerns. Therefore, the fermentation of LAB strains such as GABA-producing *Levilactobacillus brevis* is proposed as an alternative to establishing ecological processes for the production of 2-pyrrolidone [21]. During this same period, the research on sourdough for the development of GABA-enriched functional foods is highlighted [15]. In the 2018–2020 period, a convergence of the keyword combinations “*Saccharomyces cerevisiae* and glutamate decarboxylase” and “lactic acid bacteria (lab) and gamma-aminobutyric acid (GABA)” towards “γ-aminobutyric acid and *Lactiplantibacillus plantarum*” and “gamma-aminobutyric acid (GABA) and neurotransmitter” was observed which then converged to “optimization and gamma-aminobutyric acid (GABA)” for the 2022 period. These combinations highlight the focus on parameter optimization in the fermentation of LAB strains to maximize GABA production. In the period 2020–2022, convergence was found between keyword combinations such as “*Tetragenococcus halophilus* and soy sauce” towards “cheese and *Lactococcus lactis*”, demonstrating the recent trend towards isolating strains from fermented foods such as soy sauce and cheese to promote GABA elicitation [22].

## 3. GABA Producing Microorganisms

GABA synthesis from microbial species is an important source to increase the low content available in foods such as vegetables and fruits [23]. In the market of foods and beverages, there are only a few GABA-prepared products available, including tablet and capsule forms [24]. Food enrichment through the fermentation process is a commonly and widely used method. The fermentation process is related to the glutamic acid decarboxylase (GAD) production ability of microorganisms to catalyze the irreversible decarboxylation of L-glutamate to GABA [25]. The framework to identify the producing strains starts with the screening of different sources, mainly traditionally fermented foods, followed by subsequent isolation, cultivation, and genetic identification. This methodology sometimes includes quantification and optimization of GABA production.

**Figure 4:**
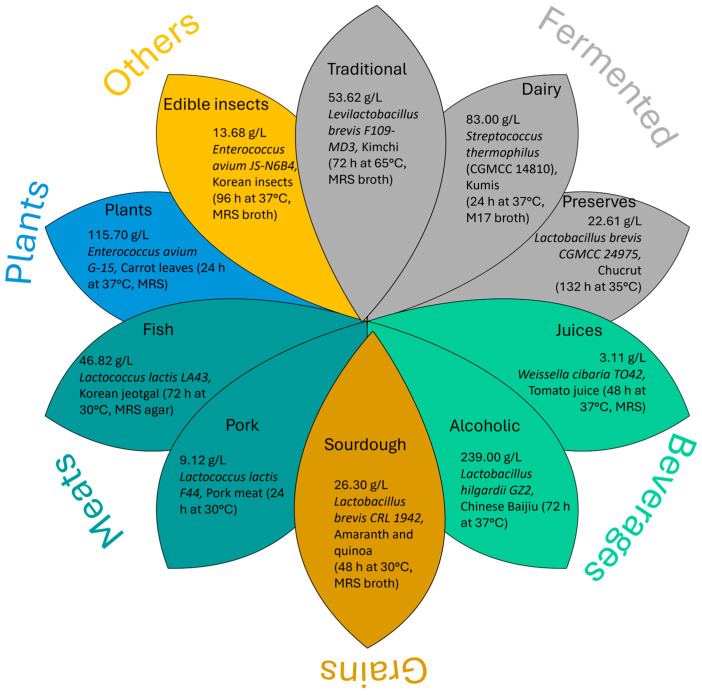
GABA-producing strains by food matrix with maximum yield, strain, food, and culture conditions.

The most common sources related to GABA-producing strains are fermented foods. One of the early reports was made by Komatsuzaki et al. [1]. In this study, NFRI 7415 *Lactobacillus* strain was isolated from traditional fermented foods (*funa-sushi*) and its maximum GABA production yield was established in 31.1 g/L at regulated pH 5.0 and 30 °C for 7 days of fermentation. They also observed the beneficial effect of adding pyridoxal 5-phosphate (PLP) on the increase in GABA accumulation during LAB fermentations. However, nowadays it has been possible to isolate/grow microorganisms with the ability to produce GABA from different food sources such as dairy products, flowers and leaves, preserved foods, dressings, beverages (juices and alcoholics), grains (whole or ground), meats (pork, fish, and seafood), and edible insects. Figure 4 lists the identified food matrices, the highest GABA yield reported, and the corresponding strain/culture conditions.

### 3.1. Production of GABA by Fungi and Yeast

Isolated strains of GABA-producing fungi and yeasts originate from diverse sources, ranging from food matrices to complex ecosystems like intestinal flora and marine environments. Aoki et al. [26] utilized *Rhizopus* strains in a mixed aerobic-anaerobic cultivation system to promote GABA accumulation in soybeans. This approach leveraged the stress-induced metabolic shift in plants, where low atmospheric oxygen inhibits GABA catabolism to succinic acid, resulting in a GABA-enriched fermented soy product. Similarly, Kadir et al. [27] investigated GABA production in soy food fermentations using *Aspergillus* strains, demonstrating the influence of physical (pH and temperature) and nutritional (carbon and nitrogen sources) conditions on GABA production. Traditional fermented vegetables also serve as substrates for GABA production. Q. Zhang et al. [28] employed *Saccharomyces* strains isolated from Chinese pao cai and co-cultured with *Lactiplantibacillus plantarum* to produce GABA-enriched mulberry brewed beverage. This beverage exhibited a complex flavor profile attributed to the presence of tartaric, succinic, lactic, malic, citric, and oxalic acids as well as some fruity esters.

Dairy products represent another important source of GABA. Co-culturing known GABA-producing lactic acid bacteria (LAB) with yeasts is a common strategy. Hurtado-Romero et al. [29] investigated autochthonous lactic acid bacteria and yeasts from artisanal Mexican milk kefir grains to select microbial starters with functional properties to produce fermented dairy products. Probiotic properties, fermentability with commercial prebiotics, GABA production potential, and clustering analyses suggest that three of twelve isolated strains can be successfully used in the design of new dairy-fermented products. On the other hand, Li et al. [30] evaluated the ratio of bacteria/yeasts on cheese fermentation regardless of free amino acids, aromas, and GABA content. The study found that mixed strain fermentation reaches higher GABA contents (189 mg/100 g) and is more beneficial to the production of cheese flavor substances. A recent study evaluated the resistance to acid, alkali, bile salts, trypsin, and pepsin of the gastrointestinal tract of beneficial microorganisms isolated from Iranian milk kefir beverages. In this study, Moghimani et al. [31] emphasizes the importance of resistance of valuable natural compounds to the gastrointestinal tract and the use of food matrices as carriers that can effectively increase the survivability of isolates in the gastrointestinal tract.

Guo et al. [32] isolated and characterized marine yeasts from various shores in Japan. *P. anomala* was identified as a GABA producer and accumulator. The authors suggest a link between its high GABA production capacity and osmotic pressure resistance, although further investigation is required. Other studies have explored diverse ecological niches, including wildflowers [33] and coconut cake byproduct from oil extraction, demonstrating the broad potential for isolating GABA-producing fungi and yeasts. On the other hand, Sun et al. [34] evaluated the production of fermented apple beverages enriched with GABA produced by *Saccharomyces cerevisiae* SC125. The authors reported a yield of 898.35 ± 10.10 mg/L of GABA through the efficient bioconversion of L-monodeoxyglutamic acid. The apple beverage with *S. cerevisiae* SC125 also showed improvements in taste, aroma, and overall acceptability. Table 2 summarizes the findings, including GABA yields reported in different studies.

### 3.2. Production of GABA by Bacteria

#### 3.2.1. Lactic Acid Bacteria

Cheeses are a common matrix both to isolate and to apply GABA-producing microorganisms. An early study conducted by Siragusa et al. [37] identified 61 species of 440 isolates from Italian cheeses that showed capacity to synthetize GABA. The authors found twelve species by partial sequencing of the 16S rRNA gene. Some Italian cheese varieties studied were Parmigiano, Regiano, Barricato SM, Vento dE, Ubriaco dR, Caciocavallo, Gorgonzola, and Crescenza, according to previous reports about the potential use of cheeses as a vehicle for GABA. Lather [38] employed Lactiplantibacillus plantarum DSM19463 isolated from those cheeses to optimize GABA synthesis reaching 291-fold higher maximum yield than Siragusa et al. [37]. However, in terms of yield per liter, Enterococcus malodoratus (MT742858, MT742859, MT742860, and MT742896) and Levilactobacillus brevis BGZLS10-17 strains studied by Coelho et al. [39] and Sokovic Bajic et al. [40], respectively, reported the highest values, up to 5.50 g GABA/L. Both authors isolated straight from artisanal and origin cheeses emphasizing the probiotic potential of these sources.

Isolated strains from cheeses were subjected to optimized growth using common LAB-formulated media such as MRS and M17. This isolation, identification, and cultivation strategy under optimal conditions has enabled the recognition of strains involved in cheese ripening processes with potential for GABA production (1.71 mg/kg cheese or 262 mg/L) in vitro. Identifying these strains and the specific cheese varieties they originate from facilitates the development of tailored starters for the dairy industry, promoting cheese production under processing conditions that enhance GABA content in these kinds of foods. Notable findings in this area include the positive effect of cheese ripening on GABA content [41], the survival rate of certain LAB strains under simulated gastrointestinal conditions [37,42], the capacity of identified *Lactobacillus* species to produce high GABA levels under unoptimized conditions [43], and the use of hard and semi-hard cheeses as suitable matrices for optimizing GABA production [44]. To integrate these outcomes with the experimental evidence, Table 3 presents a consolidated overview of the reported GABA yields, culture media, and the principal observations associated with LAB isolated from various dairy products.

Lactic acid bacteria are strongly related with fermented milk due to its facultative heterofermentative capacities (Table 3). Fermentation processes are followed by acidification (pH drop) caused by intracellular protons (H^+^) consumption during the decarboxylation of glutamate by the GAD route; Hu et al. [55] reported *Streptococcus thermophilus CS9* as a high GABA producer in yogurt and skimmed milk because it maintains the pH homeostasis under acidic conditions, reaching 950.36 mg/L in MSG supplemented M17 medium. Similarly, Tamés et al. [52] using lyophilized pellets of MRSc cultures (about 10^8^ CFU/mL) of *Bifidobacterium adolescentis* (IPLA60004) achieved an 896 mg GABA/L in a dairy matrix (semi-skimmed milk supplemented with MSG, D-Glucose, Casein hydrolysate). The authors attribute this high GABA yield to the strain’s ability to counteract pH acidification during bacterial growth in fermentation process. This effect is likely linked to the metabolic activity of *B. adolescentis,* where GABA biosynthesis via GAD helps neutralize acidification by consuming protons, thereby promoting a favorable environment for bacterial growth and GABA accumulation.

Other authors have investigated the delivery of metabolites through chocolate formulations to enhance the viability of GABA-supplemented foods. By means of microencapsulation by freeze-drying, Youssef et al. [58] added MRS broth (supplemented with MSG) with suspended *Levilactobacillus brevis* Y1 and *Lactiplantibacillus plantarum* LM2 cells to a milk chocolate drink, managing to maintain the persistence of GABA until the end of the storage period (one month). Ozer et al. [59] achieved the highest GABA yield (59.00 g/L) in the studies with dairy products, applying microencapsulated *Lacticaseibacillus rhamnosus* NRRL B-442 in chocolate.

On the other hand, Table 4 presents additional food-derived sources used to investigate microbial GABA production. Among these, traditional fermented vegetables stand out as one of the most common and productive substrates, providing diverse microbial communities and favorable conditions for identifying high GABA-producing strains. Up to 22 LAB species have been identified in Kimchi, exhibiting a wide range of GABA yields (60 to 53,000 mg/L) depending on strain, fermentation conditions, and culture supplementation. *Levilactobacillus brevis* GABA 100 is a registered strain derived from Korean kimchi known for high GABA production [16]. Four strains of *L. plantarum* in pao cai and Thai foods were cultured obtaining low (143 mg/L) [24] to high (6350 mg/L) [60] GABA yields. *Levilactobacillus brevis* CGMCC 24,975 in chucrut exhibits an up-regulated GAD gene expression that allowed the development of a batch fermentation process on a 5-L scale, laying the foundation for producing efficient food-safe GABA, according to the authors [61].

The vegetables undergo extraction, grinding, and suspension processes to obtain various liquid foods, which are used to isolate LAB and obtain GABA (Table 4). Vegetable milks are common products, such as adzuki bean milk, chickpea milk, and soymilk. The highest GABA production reported in soymilk reached 2302 mg/L using an MRS-complemented medium (MSG) for 48 h of fermentation at 37 °C. The authors concluded that one day of soybean germination can significantly increase the GABA content in the final product [110]. Xia et al. [84] studied the effects of a strain complex (*Limosilactobacillus fermentum* SMN10-3 and *Lactococcus lactis* SMN15-6) on the GABA formation, flavor, and metabolic pathways in fermented soymilk. Their findings showed a GABA yield of 1.76 mg/mL. They also identified 55 aroma and flavor-related metabolites produced after fermentation, of which 28, dominated by hexanal, were significantly downregulated, and 26, dominated by alcohols, were significantly upregulated. The significant metabolic pathways involved were d-alanine, taurine, hypotaurine, and selenocompound metabolism. Soft vegetable foods have also been investigated. Litchi and tomato juices fermented for two days at 37 °C yielded GABA contents up to 300 mg/L [83] and 3113 mg/L [87], respectively.

*Lactobacillus* and *Weissella* species are commonly isolated from plant-based matrices, including sourdoughs, plant leaves, and fermented grains. Culturing these microorganisms typically involves the use of specially formulated media supplemented with MSG to enhance GABA production. GABA yields vary significantly, ranging from 0.10 to 26.30 g GABA/L with an average fermentation time of approximately 40 h. Fermentation periods for sourdough, flours, leaves, and whole grains generally exceed 40 h. However, few studies have explored shorter fermentation periods. For example, Venturi et al. [91] reported a GABA yield of 39 mg/kg in bread after only 6 h of fermentation. Conversely, longer fermentation periods, spanning several days, can result in moderate GABA yields. Rodriguez-Sánchez et al. [94] demonstrated this with a 7-day fermentation of Almagro eggplants resulting in a relatively lower yield of 0.66 g GABA/L. Beyond plant-based matrices, the diversity of GABA-producing microorganisms extends into animal-derived fermented foods. While sourdoughs, leaves, and grains provide favorable environments for LAB with notable variations in GABA yields, fermented fish and meat products represent another important group of substrates where distinct microbial strain contribute to GABA biosynthesis (Table 4). A wide variety of fermented fish products have been documented in recent years. Traditional products such as *Jeot-gal*, *Nham*, *Plaa-som*, *Mam nem*, and *Budu fish* are commonly associated with GABA production. Similarly, a diverse range of LAB species and strains, along with various fermentation processes and culture media, have been reported in association with GABA production. Notably, fresh fish and shrimp have been identified as sources of *Levilactobacillus brevis* RK03 and *Enterococcus faecium* SH9, which have demonstrated GABA production in supplemented media. Reported GABA yields range from low levels (65.5 mg/L) [106] to moderate levels (970 mg/L) [105]. Furthermore, both fermented and stuffed pork products have been investigated, with multiple studies consistently reporting high GABA yields, reaching up to 1000 mg/L, thus highlighting the potential of GABA-enriched pork products.

On the other hand, edible insects represent a promising source of GABA-producing strains. These organisms hold potential applications in food technology, particularly as edible protein substitutes owing to their high protein content. A *Lactobacillus* strain (*plantarum* Taj-Apis362) isolated from honeycomb and the honey stomach of the Asiatic giant honeybee (*A. dorsata*) in Malaysia, exhibited high GABA-producing ability among 24 selected strains. Based on 16S rDNA sequencing and GenBank database analysis, Taj-Apis362 DSM 13,600 was assigned the accession number HM027644, belonging to *Lactiplantibacillus plantarum* [111]. These researchers employed MRS broth supplemented with 50 mM glutamic acid, achieving a maximum GABA production of 181.48 mg/L. Optimization of culture conditions, specifically glutamic acid concentration (497.97 mM), temperature (36 °C), initial pH (5.31), and incubation time (60 h), significantly enhanced GABA production, reaching 737.29 mg/L. These findings suggest that the studied strains could accelerate the development of functional fermented foods. Similarly, other researchers have utilized the same methodology to identify *Enterococcus avium JS-N6B4* from domestic Korean insects (*G. bimaculatus*, *T. molitor larvae*, *P. brevitarsis larvae*, and *A. dichotoma larvae*). Optimization by focusing on glucose, yeast and MSG concentrations resulted in 13,680 mg GABA/L, a 2.79-fold increase compared to the production achieved with basic medium [112].

Some studies have successfully isolated GABA-producing strains from singular sources. *L. planyarum* 90sk and 29sk, *L. brevis* 15f, *B. adolescentis* Tv29, km5-1, *B. angulatum* GT102, and *B. dentiuum* 9 were obtained from human microbiota (feces, saliva and vagina). In the study by Yunes et al. [113], 58 out of 135 strains, representing five species capable of producing GABA via identified *gad*B/*gad*C genes, were found. The authors highlight *Bifidobacteria* the main GABA producers among these strains. A separate study isolated *Liquorilactobacillus hilgardii* strain GZ2 from Chinese liquor (Baijiu) and optimized several culture conditions including carbon and nitrogen sources, temperature, pH, and the concentrations of MSG and glucose. Using fed-batch fermentation, they achieved an exceptionally high GABA yield of 239 mg/L after 72 h. This substantial production suggests that *L. hilgardii* GZ2 holds potential for the development of health-promoting functional foods and medical applications. Similarly, Li et al. [114] recently isolated *Enterococcus avium* (GL1), a potentially probiotic strain, from Chinese liquor (Baijiu). *E. avium* GL1 exhibited high GABA production, reaching a concentration of 206.84 g/L with a volumetric productivity of 2.87 g/L-h. This underscores the potential of unconventional sources, like fermented beverages, in the search for novel GABA-producing strains. While LAB remain the most commonly reported microorganisms for GABA synthesis, these findings suggest that non-fermented products can also serve as reservoirs for GABA-producing bacteria. Furthermore, GABA production can be significantly enhanced through optimized culture media and fermentation conditions, as evidenced by the reported yields.

Overall, GABA production during fermentation is consistently associated with specific lactic acid bacteria genera, particularly *Levilactobacillus*, *Lactiplantibacillus*, *Lacticaseibacillus*, *Enterococcus*, and *Lactococcus*, regardless of the food matrix. This pattern suggests that intrinsic microbial traits, especially the efficiency of the glutamate decarboxylase system and acid stress tolerance, play a decisive role in GABA biosynthesis. Dairy-based systems, including cheeses, milk, and yogurt, generally exhibit moderate to high and more reproducible GABA yields, supported by stable pH conditions and the availability of amino acid precursors. In contrast, plant-based fermented matrices show much greater variability, ranging from low to exceptionally high GABA concentrations, reflecting differences in substrate composition and microbial adaptation. Despite this variability, plant-derived systems often achieve higher maximum yields, particularly when dominated by *Levilactobacillus brevis* and *Lactiplantibacillus plantarum*. Meat, fish, and unconventional substrates, such as edible insects and fermented beverages, further demonstrate the versatility of GABA-producing microorganisms, although these systems remain less explored. These findings indicate that while substrate characteristics modulate GABA production efficiency, microbial genotype is the primary determinant. Consequently, integrating targeted strain selection with substrate-specific fermentation optimization is essential to maximize GABA yields and enhance the industrial applicability of fermented GABA-rich foods.

#### 3.2.2. GMO Bacteria and Fungi

In vivo mutagenesis technologies are applied to build a bio-manufacturing platform through microbial production. This strategy can attend industrial production of amino acids and bulk chemicals. Since *Corynebacterium glutamicum* is a natural L-glutamate producer, it has been used for the safe production of amino acids. GRAS (generally recognized as safe) status promotes the application of *C. glutamicum* in food and pharmaceutical-related chemicals [115]. Corresponding genes that encode GAD can be cloned and expressed using protein engineering strategies to biosynthetic production of GABA. The construction of a new GABA producing system in the whole-cell system led to achieve competitive levels of GABA production even without supplying additional pyridoxal 5′-phosphate (PLP) cofactor [116].

To avoid inhibition in GABA production by Glutamate decarboxylation, Jorge et al. [117] engineered an alternative route by heterologous expression of two enzyme genes from *Escherichia coli,* increasing GABA accumulation by 51%. Another strategy to enhance GABA accumulation during fermentation involves the genetic modification of *Corynebacterium glutamicum* to disrupt GABA transport and metabolism. Zhao et al. [118] developed the mutant strain RES167 by deleting the *C. glutamicum* GABA-specific transporter (*gabP*), achieving a 12.5% increase in GABA productivity. Similarly, Wei et al. [115] constructed a GABA-producing strain by deleting the GABA degradation pathway and introducing an exogenous GABA biosynthetic pathway, enabling GABA production from glycerol.

To further optimize metabolic flux, the authors designed a tunable, growth phase-dependent autonomous bifunctional genetic switch (GABS). This system, based on growth phase-responsive promoters and degrons, dynamically redirects carbon flux, allowing a metabolic transition from a growth mode to a production mode in *C. glutamicum* strain G7-1. Additionally, the co-utilization of multiple carbon sources has been explored to broaden the range of fermentable substrates. Buitrago et al. [119] applied this strategy to engineer *C. glutamicum* strain H36GD1852 for the efficient utilization of both glucose and xylose, enhancing GABA production from agro-industrial waste. Using degenerate primers, Fan et al. [120] inserted the *gad* gene from *Levilactobacillus brevis* CGMCC 1306 into *Escherichia coli* BL21, finding that this modification not only influenced GAD enzyme production, but also increased cell density during fermentation, enhancing GABA biosynthesis efficiency. Since GABA synthesis is catalyzed by GAD, the enzyme’s production plays a crucial role in overall GABA yield.

A key advantage of using recombinant GAD is circumventing microbial fermentation, simplifying the process to downstream purification of GABA [121]. The authors demonstrated that enzymatic GABA synthesis using purified recombinant GAD from *Lactiplantibacillus plantarum* FNCC 260 yielded 5- to 7-fold higher product concentrations compared to microbial fermentation, achieving this in considerably less time. Furthermore, Ham et al. [116] enhanced GAD activity by overexpressing pyridoxal kinase (*pdxY*) in *E. coli* K12 to improve regeneration of the essential GAD cofactor, PLP. By co-expressing *L. brevis* GAD in this engineered strain, they facilitate efficient conversion of MSG to GABA while concurrently replenishing PLP levels. This approach addresses the potential limitation of PLP availability during enzymatic GABA synthesis. Efficient cofactor regeneration is crucial for maximizing GAD activity and overall GABA production. Genetically modified strains, key enzymes, and GABA yields obtained with bacterial and fungi GMOs are summarized in Table 5.

GMO fungal strains have also been investigated in GABA production. For example, Mo et al. [122] reported the transformation of *Monascus pilosus* to enhance GABA biosynthesis through GAD overexpression. Among the transformants obtained, most showed increases of up to 134.1% in GABA compared to the parental strain. Multiome analyses revealed that GAD overexpression regulates key genes and metabolites involved in central metabolic pathways, simultaneously promoting GABA biosynthesis. Furthermore, genetic modification strategies have been extended to edible basidiomycete fungi. In a genome-guided approach, Li et al. [123] reported that the *Ff*-*gad2* gene encoding glutamate decarboxylase from *Flammulina filiformis* was heterologously expressed in *Hypsizigus marmoreus*, resulting in a substantial increase in GABA production, with increases ranging from 85.4% to 283.9% compared to the wild-type strain. This genetic modification also led to improved mycelial growth and biomass accumulation, confirming the catalytic role of GAD in fungal GABA biosynthesis. The study further suggested that genetic constructs containing introns can significantly improve heterologous gene expression in fungi, providing evidence for a promising strategy for developing fungal strains that produce high levels of GABA.

Genetic engineering in bacteria and fungi has proven to be an effective strategy for enhancing GABA production, mainly through the overexpression of glutamate decarboxylase, the elimination of competing pathways, and the optimization of metabolic and cofactor flow. Advances in bacteria such as *Corynebacterium glutamicum* and *Escherichia coli*, along with recent studies in edible and filamentous fungi, confirm that these modifications not only significantly increase GABA yields but can also improve cell growth. Taken together, these results position genetically modified microorganisms as promising platforms for efficient GABA production.

## 4. Alternatives in GABA Identification and Quantification

Structurally, GABA is a four-carbon non-proteinogenic amino acid characterized by the presence of a carboxyl (-COOH) and an amino (-NH_2_) functional group. This small molecule, with the chemical formula C_4_H_9_NO_2_ and a molecular weight of 103 Da [124], was first identified as a neurotransmitter in the mid-1970s, alongside other neuropeptides, sparking significant research interest [125].

Due to its biological relevance, numerous analytical methodologies have been developed to quantify GABA across different matrices and identify GABA producers. These approaches can be categorized into three main groups: instrumental (in vitro), computational (in silico), and in vivo techniques (outside the scope of this review). In vitro methods encompass laboratory-based analytical techniques for GABA quantification in diverse biological and food samples, while in silico approaches rely on computational tools to predict and identify microbial strains with GABA-producing potential. The following two subsections will discuss the methodologies most commonly employed in the studies considered: in vitro analytical techniques for GABA measurement, and bioinformatics (in silico) strategies used for microbial identification and metabolic pathway analysis.

### 4.1. GABA Quantification: In Vitro Methodologies

A large number of studies in the retrieved information stand out for the direct quantification of GABA content in food sources, culture media, or fortified foods. This approach to quantification is mostly applied when there is prior certainty of GABA production. In this sense, techniques such as amino acid analyzer (AAA), high performance liquid chromatography (HPLC) and its variants, capillary electrophoresis time-of-flight mass spectrometry (CE-TOFMS), liquid chromatography-mass spectrometry (LC-MS), gas chromatography-mass spectrometry (GC-MS), thin layer chromatography (TLC) and its variants, GABase enzymatic assay (spectrophotometric), and colorimetric phenol-hypochlorite method (Berthelot) have been applied. On the other hand, a presumptive approach based on early pre-assessment and subsequent quantification has also been adopted. With this approach it has been possible to analyze large numbers of strains and sources with indications of GABA production while keeping research costs down. The techniques that are most often combined are TLC for prescreening and HPLC for quantification, TLC for prescreening and GABase enzymatic assay for quantification, TLC for prescreening and colorimetric phenol-hypochlorite method (Berthelot) for quantification, preparative TLC for sample extraction and cupric-sulfate development for quantification. However, there are investigations with the objective of identifying the presence of GABA, but not of quantifying it. In these cases, the reported methodologies include qualitative TLC, colorimetric pH indicator method, colorimetric GAD assay, or gas release method. General procedures, equipment, reagents, and details are summarized in Table 6.

**Figure 5:**
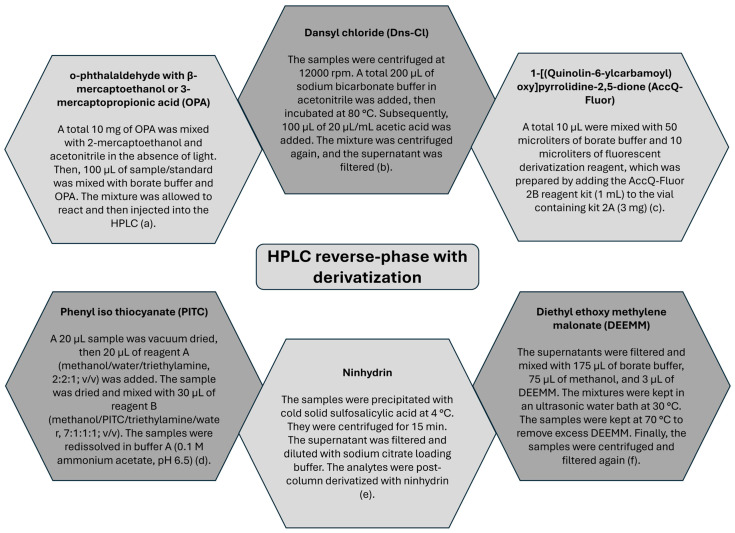
Common derivatization reagents for quantification by chromatography. Protocols by (**a**) [144], (**b**) [145], (**c**) [16], (**d**) [80], (**e**) [37], (**f**) [146]. Reagent volumes and proportions may vary according to the authors.

According to Table 6, 68% of reviewed reports that applied in vitro quantification used chromatography (HPLC or UPLC). However, due to lack of chromophore groups in GABA [143], derivatization procedure is required for detection of the molecule. The derived complex can be subsequently quantified using a standard solution and a typical calibration curve. In Figure 5, derivatizing reagents used to quantify GABA are presented, as well as the protocol applied by some authors and the wavelength for the detection of the obtained complex.

Pre-column derivatization procedures are the most reported. This may be related to the stability of the complexes and the ease of subsequent determination. o-phtaldehyde (OPA) with β-mercaptoethanol or 3-mercaptopropionic acid was used in 32% of total reports by [28,39,42,55,60,67,69,70,77,90,105,130,131,134,136,144]. Phenyl iso thio cyanate (PITC) was used in 27% of total reports by [27,30,40,53,56,57,79,81,92,97,101,106,107,111,112,147], Dansyl chloride (Dns-Cl) was used in 18% of total reports by [48,65,83,86,91,110,120,127,128,145,148], AccQ-Fluor (1-[(quinolin-6-ylcarbamoyl) oxy] pyrrolidine-2,5-dione) was used in 11% of total reports by [45,47,51,93,133,135], DEEMM (diethyl ethoxy methylene malonate) was used in 8% of total reports by [52,94,116,146] and post-column Ninhydrin derivatization was used in 5% of total reports by [35,37,38]. There are other derivatization reagents such as DABS-Cl (4-dimethylamine phenyl azobenzylsulfonyl chloride) [149], DNFB (2,4-dinitrofluorobenzene) [150], and Fmoc-Cl (9-Fluorenylmethyloxycarbonyl chloride) [143].

From a practical standpoint, the main analytical methods for in vitro GABA determination differ markedly in sensitivity, cost, and suitability for specific research objectives. HPLC/UPLC-based methods, typically coupled with pre- or post-column derivatization, offer high accuracy, reproducibility, and low limits of detection, commonly in the low micromolar range (approximately 0.1–5 µM, depending on the derivatization reagent and detector). These techniques are well-suited for definitive quantification, kinetic studies, and validation of fermentation performance, but they require costly instrumentation, derivatization steps, trained personnel, and relatively long analysis times. In contrast, TLC-based methods represent a low-cost, rapid, and accessible alternative, particularly suitable for high-throughput prescreening of large strain collections or fermentation conditions. Quantitative or densitometric TLC typically exhibits higher detection limits (approximately 50–500 µM) and lower precision than HPLC, limiting its use for accurate quantification but making it highly valuable as an initial screening tool. Enzymatic GABase assays occupy an intermediate position, combining moderate sensitivity (limit of detection approximately 5–20 µM), relatively low operational costs, and simpler workflows compared to chromatography. These assays are appropriate for routine quantification in fermentation broths when matrix interferences are controlled, although they may suffer from enzyme cost, limited specificity in complex samples, and reduced robustness compared to chromatographic techniques.

### 4.2. Bioinformatics (In Silico Tests)

The remarkable advances in bioinformatics tools have enabled the extensive application of in silico approaches for the characterization, selection, and optimization of GABA-producing microorganisms. Genomic and transcriptomic frameworks now support key research objectives, including whole-genome analysis [76], probiogenomic [50] and safety/virulence/antibiotic-resistance studies [52]. Most studies reviewed follow a standardized bioinformatics workflow comprising five main analytical steps: raw data quality assessment, preprocessing, sequence alignment, post-processing, and variant analysis, including detection, annotation, and prioritization [151].

#### 4.2.1. Genomic and Comparative Analysis of LAB Strains

A representative and comprehensive application of these approaches was reported by Surachat et al. [152], who conducted an in silico genomics analysis of *Lactobacillum plantarum* DW12. Genome assembly was performed using Canu (version 2.3), with quality evaluation by QUAST (version 5.3.0) and completeness assessment by BUSCO (version 6.0.0). Assembly graphs were visualized using Bandage (version 0.9.0), while unmapped reads were extracted using SAMtools (version 1.22.1) to identify small plasmids with SPAdes (version 4.2.0). Plasmid sequences were further analyzed using PlasmidFinder (version 2.1.6), BLASTx (version 2.16.0), and BLASTp (version 2.16.0), circularized with Circlator (version 1.5.6), and polished with Pilon (version 1.24). Genome annotation was carried out using the RAST server (version 2.0), with rRNAs and tRNAs identified by RNAmmer and tRNAscan-SE, respectively. Prophage regions and tandem repeats were predicted using PHASTER (version 2.0) and Repeat Finder (version 4.09.1), while CRISPR arrays were detected with CRISPRFinder (version 4.2.20). Transmembrane helices were predicted using TMHMM Server 2.0, and genomic islands were identified with IslandViewer 4. Integrative and conjugative elements (ICEs) were detected using ICEberg 2.0. Genome visualization was performed using Circos (version 0.69.9) and CGView (version 1.8.0).

Comparative genomic analysis retrieved 584 genomes (155 complete genomes, 186 scaffolds, and 243 contig levels) of *L. plantarum* from the GenBank database. Data obtained were then used to perform a pan-genome analysis to identify core, accessory, and unique protein families using Roary. The authors also performed a pan-genome analysis on 31 GABA-producing strains obtained from literature reviews and available genomic sequences in public databases. RiPPs and bacteriocin-encoding genes were identified by sequence similarity search using BLASTp against the Bagel database. Gene clusters of interest were then analyzed and visualized by the Bagel4 webserver (version 1.0). Also, all restriction-modification (R-M) systems were searched in all genomes by BLASTn (version 2.17.0) against the R-M genes retrieved from the National Center for Biotechnology Information (NCBI) database. Phylogenetic threes construction was developed in MEGAX software (version 10.2.6) using 640 core genes from 577 bacterial strains (including DW12) to perform multiple alignments in MUSCLE (version 5.1.0). Finally, the antibiotic-resistance genes were searched using the Resistance Gene Identifier (RGI) server (version 6.0.5). The stability of the genome was evaluated by using several tools including PHASTER, PathogenFinder (version 2.0.5), ISfinder (https://isfinder.biotoul.fr accessed on 23 January 2025), and PlasmidFinder to identify prophage, pathogenic genes, insert sequences, and plasmid sequences, respectively.

According to the findings, the authors argue that *L. plantarum* strains contain various class II bacteriocin-encoding genes (plantaricin genes). These bacteriocins provide interesting mechanisms to inhibit the growth of pathogens and do not affect eukaryotic cells. Results also showed that *L. plantarum* DW12 encoded significant pathways of GAD biosynthesis, supporting its ability to potentially synthesize GABA. Furthermore, safety assessment analysis demonstrated that there was no evidence for virulence factors or AMR genes in the DW12 genome. Thus, DW12 could be a good candidate for use as a starter culture in the food and beverage industries.

#### 4.2.2. Functional Identification of Genes Involved in GABA Transport and Production

Zhao et al. [118]. applied a similar genomic strategy to identify and experimentally validate the gene *ncgl0464* as a major GABA transporter in *Corynebacterium glutamicum* RES167. Genome sequences were retrieved from GenBank, and gene and protein sequences were obtained from NCBI and KEGG databases. Sequence similarity analyses were conducted using BLAST (version 2.17.0). Deletion of the GABA-specific transporter gene (*GabP_cg*) resulted in a 12.5% increase in GABA productivity compared to the parental strain, highlighting the importance of transporter engineering for enhancing GABA production. Similarly, Guo et al. [32] compared the aligned sequences to identify and classify four yeast isolates through GenBank databases by Basic Local Alignment Search Tool (BLAST). Then, nucleotide sequences were used in phylogenetic analysis by the neighbor-joining method performed in CLUSTALW software (version 2.1). The authors found two strains belonging to the genus *Pichia* with the highest GABA producing ability and propose to name *P. anomala* MR-1 and MR-2.

#### 4.2.3. Probiogenomic Assessments, Distribution of Gad Genes and Computational Modeling

Tamés et al. [52] performed in silico analysis to evaluate metabolic and safety traits of selected *Bifidobacterium adolescentes* strains. Summarized methodology was genome sequencing at GenProbio S.R.L. The genome comparisons were made against a genome template of *B. adolescentis* deposited in the NCBI database (*B. adolescentis* LMG10502 strain, GenBank assembly accession GCA_000010425.1). Quality control of sequencing reads was performed using the fastq-mcf (v.1.04.807) tool to scan sequence files for adapters, skewing detection, and quality filtering. Then, preprocessed reads were assembled using SPAdes (v.3.15.5), and genome contig reordering based on their sequence length was performed using Bwa (v.0.7.17-r1188) and Samtools (v. 1.6.0). Genome assemblies of *B. adolescentis* strains were annotated following complementary pipelines. In this sense, antibiotic resistance genes (ARGs) were annotated using TORMES pipeline v.1.3.0. For this purpose, genome assemblies were mapped against Resfinder and Comprehensive Antibiotic Resistance Database (CARD), both databases implemented in TORMES pipeline. In addition, genome sequences were mapped against the Virulence Factors Data Base (VFDB) database to identify the presence of virulence genes [153]. Prodigal (v.2.6.3) software for gene prediction was used to determine open reading frames (ORFs), which were subsequently annotated by HMMER software (v.3.3.2) for biosequence analysis using profile hidden Markov models (HMMs) and Pfam database. Finally, genome assemblies were mapped against the Carbohydrate-Active enZYmes Database (CAZy, using “run_dbcan” software (version 5.2.1). This tool integrates HMMER software to annotate bacterial carbohydrate active enzymes. Only glycosidase domains showing coverage values higher than 0.95 were chosen to ensure the quality of the data generated. Further statistical analyses were performed on R (v.4.1.1). Bifidobacterial genomes were grouped according to their Pfam domains associated with sensitivity to oxygen, aerotolerance, acid tolerance, acid- and bile-stress, and CAZy domains through hierarchical clustering. These clusters were calculated by the complete linkage method using the basic function “hclust” from the R v.3.6.2 programming environment [52].

Below are other authors who performed genomic sequence analyzes and phylogenetic reconstructions. Anussara Ratanabure [97] used blastn. CLUSTALx2.0 for multiple alignments sequence comparisons with the GenBank database in the NCBI website for *Lactobacillus namurensis* NH2 and *Pediococcus pentosaceus* NH8 and MEGA4 for phylogenetic tree. Hu et al. [55] performed the whole genome sequencing of CS5, CS9, CS18, and CS20 *Streptococcus thermophilus* strains using a combined sequencing platform of Illumina platform and Pacbio RSII. Illumina PE library and PacBio library. The family distribution, conserved domain, and model structure of glycosyltransferase (GTF) were analyzed using the carbohydrate-active enzymes database, the Conserved Domain Database, and the SWISS-MODEL server. The virulence genes in the *S. thermophilus* strains were predicted based on the VFDB (Virulence Factors of Pathogenic Bacteria) database. Li et al. [114] searched similarities of the 16S rRNA gene to the type/reference strain database using the nucleotide basic local alignment search tool available at NCBI and Pacific Biosciences Sequel IIe technology (PacBio, Menlo Park, CA, USA), respectively. BothpPhylogenetic tree analysis was performed using the neighbor-joining method by MEGA11. Phuengjayaem et al. [77] developed a comparative genome sequences analysis in EzBiocloud server for sequence similarity values between the isolates (*Lactiplantibacillus plantarum* LSI2-1) and related reference strains. The phylogenetic tree based on the neighbor-joining (NJ) method and GAD genes tree based on the maximum likelihood were constructed using MEGA 7. Chintakovid et al. [50] performed a thorough silico safety assessment to support the phenotype analysis. This assessment ensured that the *Lactiplantibacillus plantarum* SPS109 strain poses no risks or concerns when employed in food-related applications as a Lab GABA-producing and cholesterol-lowering probiotic strain.

Finally, Yunes et al. [113] identified the genes involved in GABA synthesis and transport by means of a catalog of amino acid sequences of proteins encoded by gadB and gadC genes created using NCBI protein database. The catalog was built based on the genome sequences of bacteria isolated from the human gut and consists of 57 orthologues of GAD protein of 21 genera and 44 orthologues of glutamate/gamma-amino butyrate antiporter of 16 genera. A python (2.7) script ‘Neurohunter’ based on BLASTx al gorithm was created for the identification of GAD and glutamate/gamma-amino butyrate antiporter proteins/genes and the species they belong to in genomes and metagenomes. Authors found that the identified genes were found in the following genera of bacteria: *Bacteroidetes* (*Bacteroides*, *Parabacteroides*, *Alistipes*, *Odoribacter*, *Prevotella*)*, Proteobacterium* (*Esherichia*), *Firmicutes* (*Enterococcus*), *Actinobacteria* (*Bifidobacterium*). These data indicate that gad genes as well as the ability to produce GABA are widely distributed among *lactobacilli* and *bifidobacteria* (mainly in *L. plantarum*, *L. brevis*, *B. adolescentis*, *B. angulatum*, *B. dentium*) and other gut-derived bacterial species.

Computational modeling was performed by Sanchart et al. [134] to obtain the molecular model of GAD from *L. futsaii* CS3 by homology modeling via the SWISS-MODEL server. The structure of GadB from *E. coli* at low pH (PDB ID: 1PMM) was selected as the template for model building. Sequence alignment was performed using ClustalX. The resulting model was evaluated with the SWISS-MODEL server. According to the authors, the modeled structure of *L. futsaii* CS3 GAD consists of 1410 bp encoding a polypeptide of 469 amino acids with a predicted molecular weight of 53.64 kDa and an isoelectric point of 5.56. The model was based on the sequence of Lab gad genes and should be useful for future work with the enzyme. Finally, two studies related to mathematical optimization of GABA production by *Levilactobacillus brevis* CGMCC1306 and *Monascus sanguineus* fungi were found. Huang et al. [46] included an artificial neural network (ANN) and particle swarm optimization (PSO) models for the optimization of culture conditions (pH, temperature and iron sulphate heptahydrate concentration) of *L. brevis* CGMCC1306. The authors achieved the highest GABA yield of 90.57 mM under the optimized conditions predicted by a combined model of ANN and PSO that exhibits good predictability and accuracy. Dikshit and Tallapragada [35] optimized GABA yield by Plackett–Burman and response surface methodology (RSM) experimental designs and a non-statistical model using artificial neural network methodology. Maximum yield predicted from the RSM model was 15.53 mg/gds with an MSG concentration of 0.05 g at pH 7.5 and an incubation period of 20 days. The findings suggest that *M. sanguineus* culture in coconut oil cake as a substrate is an economical method with potential to obtain GABA-enriched functional food for human consumption.

## 5. GABA Metabolism

**Figure 6:**
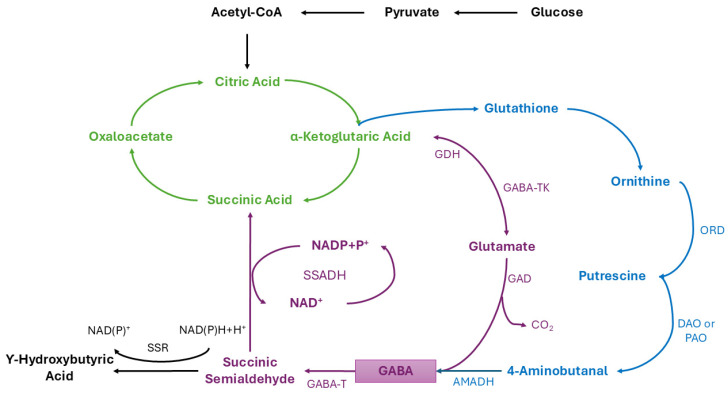
Metabolic pathway of GABA synthesis and degradation. **↑** TCA cycle, **↑** GABA shunt, **↑** PA pathway, and **↑** Degradation of GABA to GHB. Adapted from [155].

GABA synthesis occurs in the cytoplasm through two main pathways, known as the GABA shunt (or derivatization) pathway and the polyamine (PA) degradation pathway. The GABA derivatization pathway originates from the tricarboxylic acid (TCA) cycle. This pathway is used to synthesize and maintain optimal GABA levels and is considered the main synthesis pathway (70% of total GABA). Three enzymes are involved in the GABA derivatization pathway: cytoplasmic glutamate decarboxylase (GAD), GABA transaminase (GABA-T), and succinic acid semialdehyde dehydrogenase (SSADH). In this pathway, glutamate is produced from α-ketoglutaric acid. Subsequently, cytoplasmic L-glutamate undergoes irreversible decarboxylation at the α-site to produce GABA, catalyzed by GAD [154]. It should be noted that this step is the rate-limiting stage of GABA synthesis and results in the release of a CO_2_ molecule and the consumption of a proton (Figure 6).

After GABA formation, succinic acid can be generated through the action of GABA-T, which converts GABA into succinic semialdehyde; this intermediate is then oxidized to succinate by SSADH, allowing succinate to re-enter the TCA cycle [156]. On the other hand, the PA pathway involves polyamines such as spermidine, spermine, and putrescine. During polyamine degradation, intermediate compounds are catalyzed by amino oxidases, including polyamine oxidase (PAO) and diamine oxidase (DAO). This pathway produces GABA through the action of 4-aminobutanal dehydrogenase (AMADH) on 4-aminobutanal. It should be noted that AMADH, PAO, and DAO play key roles in polyamine degradation. GABA accumulation can be affected by adverse environmental conditions, such as salt stress, which increase polyamine content and consequently influence polyamine degradation [156]. GABA can also serve as a precursor for the synthesis of gamma-hydroxybutyrate (GHB), a compound produced from GABA catabolism. GHB synthesis is mediated by the action of succinate semialdehyde reductase (SSR) on succinic semialdehyde. Three genes, named SlGABA-T2, SlSSR1, and SlSSR2, are involved in this process; SlGABA-T2 regulates the conversion of GABA to succinic semialdehyde in the cytoplasm, while SlSSR1 and SlSSR2 encode SSR enzymes [157]. In microorganisms, GABA metabolism serves a dual function: as biosynthetic pathway and stress response mechanism. Cytosolic decarboxylation of L-glutamate, catalyzed by glutamate decarboxylase (GAD), is central to GABA derivation and represents the main pathway for GABA accumulation during fermentation. This reaction is physiologically relevant because it consumes intracellular protons, thereby contributing to pH homeostasis under acidic conditions and improving microbial survival. Consequently, the activity of the GAD system is tightly regulated by environmental factors such as pH. In general, GABA accumulation reflects a dynamic balance between biosynthesis, transport, and catabolism. This metabolic integration explains the strain-dependent variability observed in fermentation systems and highlights the importance of controlling environmental and nutritional parameters to promote GABA accumulation rather than degradation. Understanding these regulatory mechanisms is essential for optimizing microbial fermentation processes aimed at producing GABA-enriched foods and biotechnology products.

## 6. Trends in the GABA Market

The global GABA market is commonly categorized according to production methods, namely biological fermentation and chemical synthesis. Although chemical synthesis has historically been used for GABA production, this approach involves multiple reaction steps, high production costs, hazardous reagents, and the generation of undesirable by-products, which collectively limit its scalability and commercial attractiveness. In contrast, microbial fermentation has emerged as the preferred production route, creating significant opportunities for innovation in strain development, process optimization, and sustainable manufacturing of food- and pharmaceutical-grade GABA. This shift is underpinned by extensive evidence demonstrating the capacity of lactic acid bacteria to produce GABA efficiently and safely, as well as by advances in fermentation strategies—such as controlled pH, targeted substrate supplementation, and optimized process timing—that significantly enhance metabolic yields. Moreover, the growing demand for consistent product quality and regulatory compliance is supported by robust analytical frameworks, particularly chromatography-based quantification methods, which enable precise monitoring, standardization, and quality assurance throughout the production process, thereby facilitating the translation of fermentative GABA production from laboratory-scale systems.

Market growth is driven by the expanding use of GABA in healthcare, pharmaceuticals, functional foods, beverages, animal nutrition, and dietary supplements [158,159]. In 2024, the pharmaceutical sector accounted for 38.2% of global demand, reflecting the increasing interest in GABA-based interventions for neurological health, stress reduction, and sleep regulation. The global GABA market is projected to reach approximately USD 143.3 million by 2033, with a compound annual growth rate (CAGR) of 5% from 2023 to 2033. North America led the market in 2024, accounting for 46.6% of total sales, supported by advanced biotechnological infrastructure, stringent quality and regulatory frameworks, and growing consumer demand for mental health-oriented product infrastructure [7]. However, emerging opportunities are increasingly evident in Asia-Pacific and Latin American markets, where rising health awareness and favorable fermentation-based manufacturing capabilities are expected to accelerate market penetration.

Beyond conventional GABA supplements, one of the most promising emerging opportunities lies in the integration of GABA production within the rapidly expanding probiotics and postbiotics market. Probiotic-mediated biosynthesis of GABA represents a sustainable and value-added strategy, particularly in the context of functional foods and beverages [160]. The global probiotics market was valued at USD 44.05 billion in 2023 and is projected to reach USD 84.60 billion by 2032, growing at a CAGR of 7.40%. This growth creates substantial opportunities for the development of GABA-enriched products using selected probiotic strains capable of in situ GABA production, thereby enabling multifunctional formulations that combine gut health benefits with neuroactive properties.

In this context, postbiotics, defined as bioactive compounds produced during microbial fermentation, are gaining increasing regulatory and industrial attention due to their enhanced stability, safety, and ease of standardization compared to live probiotics. GABA-producing strains and their metabolites are therefore positioned as attractive candidates for next-generation functional ingredients, particularly in applications where shelf life, thermal stability, and regulatory compliance are critical. This shift opens new market opportunities for food manufacturers seeking clean-label, non-viable bioactive ingredients with scientifically supported health claims.

Technological innovation further reinforces these emerging opportunities. Advances in metabolic engineering, strain selection, and fermentation control have enabled higher GABA yields, reduced production costs, and improved product consistency. These developments create space for academic–industry partnerships, startup-driven innovation, and patentable fermentation platforms. Leading companies such as Ningxiang Jiayuan Biology Technology Co. (Changsha, China), Pharma Foods International Co. (Kyoto, Japan), Pfizer Inc. (New York, NY, USA), Bayer AG (Leverkusen, Germany), and Nestlé Health Science (Epalinges, Switzerland) are actively expanding their portfolios [7,161,162], while probiotic-focused companies including CHR Hansen (Hørsholm, Danmark), Lallemand (Montreal, QC, Candan), Yakult Honsha Co. (Tokyo, Japan), and Kerry Group (County Kerry, Ireland) are driving innovation at the intersection of microbial biotechnology and functional nutrition [163]. Overall, the future growth of the GABA market will be increasingly shaped by the convergence of sustainable fermentation technologies, the expansion of functional and mental health-oriented products, and the strategic integration of GABA into probiotic and postbiotic formulations. These factors collectively define a set of emerging market opportunities that extend beyond traditional supplement applications and position biologically derived GABA as a key bioactive compound in next-generation health and nutrition markets.

## 7. Conclusions

Research on fermentative GABA production has increased over the past five years. This indicates that the scientific sector is driving the development and production of GABA through fermentation-based processes. Bibliometric analysis also showed that, in recent years, studies aimed at promoting GABA production have focused on the isolation of strains from fermented foods such as soy sauce and cheese. It was also identified that the supply of GABA and GABA-fortified foods remains a major challenge for the growing GABA market. Regarding the culture conditions, it was found that the supplementation of the culture medium with carbon, nitrogen, and monosodium glutamate, among other nutrients, as well as factors such as pH, temperature, and fermentation time, influence the metabolic yield of GABA.

Advances in in silico tools, including whole-genome sequencing, pan-genome analysis, and metabolic pathway reconstruction, provide a critical bridge between fundamental microbiology and industrial implementation. These approaches enable strain selection, safety assessment, and targeted metabolic engineering, reducing development time and facilitating the design of starter cultures suitable for large-scale applications. Furthermore, the integration of statistical modeling and artificial intelligence-based optimization has proven effective in maximizing GABA yields and improving process efficiency. The review also presented analytical techniques for in vitro GABA quantification, among which TCL (for screening) and HPLC (for quantification) were the most widely used. Despite remaining challenges related to scale-up, process control, and economic feasibility, strategies such as co-cultivation, use of food-grade and low-cost substrates, and controlled fermentation systems support the transition from laboratory-scale research to industrial production. Overall, the integration of advanced analytical methods, bioinformatics-guided strain development, and process optimization positions fermentative GABA production as a scalable and practical technology capable of meeting the growing demand for GABA-enriched functional foods and nutraceutical products.

Despite these advances, critical research gaps remain. Robust and well-controlled psychobiotic clinical trials are still needed to validate the health benefits of GABA-producing strains in humans. Moreover, systems-level metabolic engineering approaches integrating multi-omics data are required to enhance strain performance and enable the use of sustainable, low-cost substrates. Furthermore, the development of cost-effective downstream processing strategies for GABA recovery and purification remains a major bottleneck for industrial implementation.

## Figures and Tables

**Table 1:** Documents with the most citations on fermentative GABA production.

Title	Journals	Number of Citations	Number of Citations per Year	References
γ-Aminobutyric acid production by culturable bacteria from the human intestine	*Journal of Applied Microbiology*	925	66.07	[12]
Production of gaba (γ-aminobutyric acid) by microorganisms: a review	*Brazilian Journal of Microbiology*	423	30.21	[8]
Production of γ-aminobutyric acid (GABA) by *Lacticaseibacillus paracasei* isolated from traditional fermented foods	*Food Microbiology*	384	18.28	[1]
Production of Gamma-Aminobutyric Acid from Lactic Acid Bacteria: A Systematic Review	*International Journal of Molecular Sciences*	293	48.66	[9]
Use of sourdough fermentation and pseudo-cereals and leguminous flours for the making of a functional bread enriched of γ-aminobutyric acid (GABA)	*International Journal of Food Microbiology*	225	14.06	[15]
Production of γ-aminobutyric acid in black raspberry juice during fermentation by *Levilactobacillus brevis* GABA100	*International Journal of Food Microbiology*	222	13.05	[16]
Production of yogurt with enhanced levels of gamma-aminobutyric acid and valuable nutrients using lactic acid bacteria and germinated soybean extract	*Bioresource Technology*	205	10.78	[17]
Production of gamma-aminobutyric acid by *Levilactobacillus brevis* NCL912 using fed-batch fermentation	*Microbial Cell Factories*	203	12.68	[18]
Antioxidant and antihypertensive properties of liquid and solid state fermented lentils	*Food Chemistry*	190	14.61	[19]

**Table 2:** GABA production related fungi and yeasts.

Type	Strain	Culture Conditions	Associated Food/Source	Yield/Comments	References
Fungi	*Rhizopus microsporus* var. *oligosporus* IFO 8631	25 h at 30 °C	Fermented soybeans (Tempeh)	The highest GABA content was 1500–1740 mg/100 g of dry fermented soybeans.	[26]
	*Monascus sanguineus* (*Monascus* sp.)	20 days at 30 °C	Coconut oil cake (agri-waste)	The maximum yield predicted from the RSM model was 15.53 mg/gds.	[35]
	*Aspergillus oryzae* NSK, NSZ, NSJ y NST similar to the reference *A. oryzae* FRR 1675	5 days at 30 °C	Soy sauce (koji)	The highest GABA concentration was obtained from NSK (194 mg/L).	[27]
Yeast	*Pichia anomala* Mccl 209/2k, *Pichia anomala* Isolate ST 3352/2-03, *Pichia anomala* (MR-1 and MR-2), *Pichiaguilliermondii* strain HK58-2	30 days at 25 °C	Marine water	Isolates Hach No. 6 and Inub No. 11 with high GABA production, Inub No. 11, corresponded to new strains of *P. anomola*.	[32]
	*Pichia silvicola* UL6-1 and *Sporobolomyces carnicolor* 402-JB-1	30 h at 30 °C	Korean wildflowers	*P. silvicola* UL6-1 with a maximum GABA yield of 136.5 μg/mL and 200.8 μg/mL from *S. carnicolor* 402-JB-1.	[33]
	*Saccharomyces cerevisiae* SC125	96 h at 30 °C	Sichuan Pao Cai	898.35 ± 10.10 mg/L of GABA	[34]
Yeast/LAB	Co-cultured *Saccharomyces cerevisiae* SC125, *Lactiplantibacillus plantarum* BC114	72 h at 30 °C	Mulberry beverage and fermented vegetables (pao cai)	Yield of 2.42 g/L of GABA. Co-cultivation promotes GABA production.	[28]
	*Enterococcus faecium* AB157 and *Saccharomyces cerevisiae* SC125	96 h at 35 °C	Sichuan pao cai	The use of co-culture increased GABA production from 1.74 g/L to 2.17 g/L.	[36]
	*Lactococus lactis* BIOTEC006, BIOTEC007, and BIOTEC008; *Kluyveromyces lactis* BIOTEC009; *Lactobacillus pseudomesenteroides* BIOTEC012; and *Leuconostoc kefiri* BIOTEC014	48 h at 30 °C	Mexican milk kefir grains	The average capacity of the strains evaluated to produce GABA is comparable to that of commercial probiotics.	[29]
	*Saccharomyces cerevisiae* DL6–20 and *Kluyveromyces marxianus* B13–5. Co-cultured with commercial starter YO-MIX187 (*Streptococcus salivarius* ssp. *Thermophilus* and *Lactobacillus delbrueckii* ssp. *bulgaricus*; Danisco A/S)	50 days at 28 °C	Cheese	The highest GABA content was 0.189 g/100 g reached in the late ripening period of cheese.	[30]
	*Enterococcus faecalis* (Accession number PP790751), *Lactococcus lactis*, (Accession number PP826201) and *Pichia fermentans* (Accession number PP803455)	48 h a 37 °C	Iranian milk kefir beverages	A qualitative approach using TLC showed that *P. fermentans* produced high levels of GABA.	[31]

**Table 3:** GABA-producing LAB isolated from dairy products.

Source	Strain	GABA Yield	Key Findings	Reference
Cheeses	*Lacticaseibacillus paracasei* PF6, *Lactobacillus delbrueckii* subsp. *bulgaricus* PR1, *Lactococcus lactis* PU1, *Lactiplantibacillus plantarum* C48, and *Levilactobacillus brevis* PM17	1.71 mg/kg (mean)	Three *Lactobacillus* strains synthesized GABA under simulated gastrointestinal conditions.	[37]
	*Lactiplantibacillus plantarum* DSM19463	498.06 mg/L	GABA induces the expression of certain human genes involved in skin protection.	[38]
	*Lacticaseibacillus paracasei* 15C, *Streptococcus thermophilus* 84C, and *Lacticaseibacillus rhamnosus* 21D-B	80.0 ± 2.7 mg/kg (highest)	Cheeses made from raw cow’s milk showed higher amounts of GABA at the end of maturation (71%).	[41]
	*Levilactobacillus brevis* BGZLS10-17	5800 mg/L	The strain BGZLS10-17 could modulate the immune response in various inflammatory diseases.	[40]
	*Lactococcus lactis* ssp. or *L. lactis ssp*. biovar *diacetylactis*. Strains ULAAC-A13 and ULAAC-A23	10–97 mg per 30 g of cheese	Hard and semi-hard cheeses are suitable matrices for GABA production.	[44]
	*Lactiplantibacillus plantarum* L2A21R1	936.8 mg/L	*Lactobacillus* strains isolated from cheese could produce high amounts of GABA.	[43]
	*Levilactobacillus brevis* DSM32386	262.06 ± 15.42 mg/L	The strain *Lb. brevis* DSM 32,386 performed well in vitro tests.	[42]
	*Lactococcus lactis* (L-571 y L-572)	1147.4 ± 6.7 mg/L by co-fermentation L-571/L-572	Milk fermented with L-571 showed the highest GABA production with 3 g/L of glutamate.	[45]
	*Enterococcus malodoratus* (MT742858, MT742859, MT742860, and MT742896)	16,400 mg/L (highest)	The tentative use of *E. malodoratus* strains as probiotics still requires further safety testing.	[39]
Milk	*Levilactobacillus brevis* CGMCC1306	9385.92 mg/L	GAD activity varied during the different phases of cell growth. Maximum activity was reached at 60 h (stationary phase).	[46]
	*Streptococcus salivarius* subsp. *Thermophilus* Y2	7984.75 ± 293.33 mg/L	Optimal temperature (40 °C) and optimal pH (4.5) for GAD reaction activity in cell biotransformation.	[13]
	*Lactiplantibacillus plantarum* NDC75017	314.56 mg/100 g	The optimal factors for GABA production were L-MSG at 80 mM, PLP at 18μM, and a culture temperature of 36 °C	[47]
	*Levilactobacillus brevis* NPS-QW-145	314.97 ± 14.45 mg per 1 kg of fermented milk	Two glutamic acid decarboxylase (GAD) genes, *gadA* and *gadB*, were found in high GABA-producing *L. brevis* NPS-QW-145.	[48]
	*Lactococcus lactis* 01-7	120 mg/L	The levels of six angiotensin-converting enzyme (ACE) inhibitor peptides were higher in GABA-rich fermented milk.	[49]
	*Lactiplantibacillus plantarum* SPS109	-	Two related genes were identified, one responsible for GABA production and the other bile tolerance (cbh)	[50]
	*Lacticaseibacillus rhamnosus* SMM37	2010 mg/L(highest)	The activity of GAD on *L. rhamnosus* was stimulated by the presence of PLP in skim milk.	[51]
	*Bifidobacterium adolescentis* IPLA60004	896 mg/L	Higher GABA producers may better counteract pH acidification occurring during bacterial growth and milk fermentation	[52]
	*Enterococcus faecium* MDM21 and *Weissella confusa* MDM8	136 mg/L final product (highest)	First report to identify *Weissella confusa* as a GABA-producing strain. New fermented milk with high GABA content was produced.	[53]
Yogurt	*Levilactobacillus brevis* M4	395 mg/L	The nutrient sources required include the primary sources of carbon, nitrogen (including GABA substrate, glutamic acid), and minerals	[54]
	*Streptococcus thermophilus* (CS5, CS9, CS18, and CS20)	950.36 mg/L	High-yield GABA strain CS9 has a high survival rate under acid treatment in medium with MSG.	[55]
	*Lactiplantibacillus plantarum* NDC 75017	273.45 ± 1.56 mg/100 g (highest)	The viability of strains upon freeze drying was strain dependent, and it is not a sufficient predictor for strain functionality	[56]
	*Enterococcus faecium* BS5	>30 mg/mL	*Ent. faecium* BS5 was resistant to acid stress, bile salt, and antibiotics.	[57]

**Table 4:** GABA-producing microorganisms isolated from food matrices.

Source	Strains	GABA Yield	Key Culture Conditions	References
Fermented vegetables	*Levilactobacillus brevis* BJ20, GABA 100, NPS-QW-(145, 171, 177, 193, 216, 242, 55, 267, 281), 877G, Y7 (OL587486), FBT215 (OL587487), MCM4, Y8, *Lactiplantibacillus plantarum*, K255, CWQ-7, *Lactococcus lactis* subsp. *Lactis* K337, BRM3, *Lactobacillus* sp. OPK 2-59, *pentosus* 9D3, *Latilactobacillus sakei* 795, HU-C2W, *Enterococcus faecium* JK29, *Levilactobacillus brevis* F109-MD3, *Pediococcus pentosaceus* JC30, *Lactiplantibacillus plantarum* Lp3, *Leuconostoc mesenteroides* K1501 and K1627	60.86–53,622.4 mg/L	MRS broth, MRS + MSG (1, 10%), M17 agar MRS + PLP	[62,63,64,65,66,67,68,69,70,71,72,73,74]
	*Lactiplantibacillus plantarum* BC114, CWQ-7	3450 mg/L, 6350 mg/L	MRS + MSG	[60,75]
	*Liquorilactobacillus pentosus* 9D3, *Lactiplantibacillus plantarum* subsp. *Plantarum* LSI2-1	143 mg/L, 22,940 mg/L	MRS broth	[24,76,77]
	*Levilactobacillus brevis* CGMCC 24975	22610 mg/L	MRS + PLP + B6 Vit.	[61]
	*Bacillus cereus* OPWW1, *Lactiplantibacillus plantarum* IFK- 10 and *Pediococcus pentosaceus* IFK-11,	523.74–3393.02 mg/L, 2680 mg/L	MRS broth	[78,79]
Fermented vegetable beverages	*Lacticaseibacillus rhamnosus* GG	1120 mg/L	MRS broth (36 h at 37 °C)	[80]
	*Lactiplantibacillus plantarum* DW12	1690 mg/L	MRS + MSG (24 h at 30 °C)	[81]
	*Lactiplantibacillus plantarum* M-6	484.41 mg/L	MRS + MSG (48 h at 37 °C)	[82]
	*Levilactobacillus brevis* (LBG-29, LBG-24, LBD–14)	>300 mg/L	MRS + MSG (48 h at 37 °C)	[83]
	*Limosilactobacillus fermentum* SMN10-3 and *Lactococcus lactis* SMN15-6, *Levilactobacillus brevis* NPS-QW 145	1760 mg/L, up to 2302 mg/L	Formulated soymilk (1.80% protein, 0.70% fat, 5.00% sucrose (added), 3.80% hydrosulose, 1.10% raffinose, 0.50 ‰ K, 0.30‰ P, 0.10‰ Ca, 0.01‰ Na) (24 h at 37 °C), MRS + MSG (48 h at 37 °C)	[84]
	*Lactiplantibacillus plantarum* L42g	496.7 mg/L	MRS + MSG (18 h at 30 °C)	[85]
	*Lacticaseibacillus casei* (ATCC 393) or *Bacillus subtilis* (ATCC 23857)	1024.5 mg/L	Tryptic Soy Broth (72 h at 37 °C)	[86]
	*Weissella cibaria* TO42	3113 mg/L	MRS + MSG (48 h at 37 °C)	[87]
Sourdough, flours, leaves or whole grains	*Enterococcus* (E.) *avium* G-15	115.70 g/L	L-MSG added (24 h at 37 °C)	[88]
	*Levilactobacillus brevis* CRL 1942	26.30 g/L	MRS + MSG (48 h at 30 °C)	[23]
	*Lactobacillus ramnosus* SP1, *Lactobacillum plantarum* T6B10	100 mg/L	MRS + cycloheximide (20 h at 30 °C)	[89]
	*Weissella paramesenteroides* N-7, *Levilactobacillus brevis* A7 and *Lactobacillus farciminis* A11	19.00 g/L, up to 39 mg/kg bread	MRS + MSG (96 h at 30 °C), MR3i broth into wheat and amaranth flour doughs (6 h at 30 °C)	[90,91]
	*Lactiplantibacillus plantarum* 8014	160 mg/L	MRS + MSG (48 h at 33 °C)	[92]
	*Lactobacillus* GTL 79	324.07 mg/L	MRS + MSG (48 h at 37 °C)	[93]
	*Levilactobacillus brevis* Lb86 and *Lactiplantibacillus plantarum* 93	663.4 mg/L	MRS broth (7 days at 30 °C)	[94]
	*Weissella confusa* G2	246.2 mg/L	MRS agar supplemented with calcium carbonate (optimized 34 h at 29.1 °C)	[95]
Meat and fish products	*Lacticaseibacillus paracasei* NFRI 7415, *Latilactobacillus sakei* A156, *Lactobacillus namurensis* NH2, 37d and 32c, *Pediococcus pentosaceus* NH8, MN12, *Lactobacillus futsaii* CS3 y CS5, *Lactobacillus farciminis* D323, *Lactiplantibacillus plantarum* L10-11, 45a, 44d and 37e, *Lactobacillus futsaii* (32d), *Lactococcus lactis* LA43, *Levilactobacillus brevis* PM03, YG331, *LentiLentilactobacillus parabuchneri* IB2C, *Leuconostoc* NC5	31.14; 0.005; 9.06; 0.006; 19.94; 15.74; 2.09; 2.87; 46.82; 0.11 and 22.50 g/L	MRS + MSG (7 days at 30 °C, 72 h at 37 °C, 48 h a 30 °C, 72 h a 45 °C, 60 h a 37 °C), MRS broth, MRS and glucose yeast extract polypep ton (GYP) broth (6 days at 25 °C), commercial mesophilic starter Lyofast MWO030 (18 h at 37 °C)	[96,97,98,99,100,101,102,103,104]
	*Levilactobacillus brevis* RK03, *Enterococcus faecium* SH9	0.06–0.97 g/L	GM broth + PLP + MSG (88 h at 30 °C)	[105,106]
	*Pediococcus pentosaceus* HN8 and *Lactobacillus namurensis* NH2, *Lactococcus lactis* F44, *Lactiplantibacillus plantarum* VL1	4.05 g/kg, 9.12 g/L, 1.10 g/g	MRS + MSG (96 h a 30 °C), GM17 medium (M17 0.5% glucose) (24 h a 30 °C), 3 days at 30 °C	[107,108,109]

**Table 5:** Genetically modified GABA-producing bacteria and fungi.

Type	Strain	Catalyzing Enzyme	GABA Yield	Reference
Bacteria	*Corynebacterium glutamicum* ATCC13032	Putrescine transaminase and Gamma-aminobutyraldehyde dehydrogenase	8.00 g/L	[117]
	*Corynebacterium glutamicum* RES167 (mutant RES167/pGXKZ9)	Glutamate decarboxylase (GAD)	23.60 g/L	[118]
	*Corynebacterium glutamicum* G7-1	GAD from automatically adjusted pathway expression	45.60 g/L	[115]
	*Corynebacterium glutamicum* H36GD1852	Mutated version of *E. coli* GAD	35.47 g/L	[119]
	*Escherichia coli* BL21 with gad gene cloned from *L. brevis* CGMCC 1306	Recombinant enzyme expressed in *E. coli* to catalyze α-decarboxylation of L-sodium glutamate into GABA	-	[120]
	*Escherichia coli* T7 with *gad*B gene from *L. plantarum* FNCC 260	Purified recombinant glutamate decarboxylase (GAD) from *L. plantarum* expressed in *E. coli*	1.23 g/L	[121]
Fungi	*Monascus pilosus*	Glutamate decarboxylase (GAD)	1600 mg/kg	[122]
	*Hypsizigus marmoreus*	Glutamate decarboxylase (GAD)	3274.43 mg/kg	[123]

**Table 6:** Documented instrumental techniques for GABA quantification.

Technique	General Procedure	Details	Reference
Automathic chromatography: AAA (Amino acid analyzer)	Dilution of the sample in buffer solution at low temperature. Supernatant with water-soluble fractions/salt filtered and injected into the analyzer	Model: LC-11A Yanako Ltd. (Kyoto, Japan); L-8900, Hitachi High-Technologies Co. (Tokyo, Japan); Biochrom 30 series, Biochrom Ltd. (Cambridgeshire, UK); JEOL Auto Amino Acid Analyzer JLC-500/V2 (JEOL, Tokyo, Japan).	
			[26,62,63,126]
			[89]
			[43,98]
HPLC (High performance liquid chromatography) with ion exchange chromatography	Samples diluted in buffer at a known pH. Suspension under gentle agitation (150 rpm) and centrifuged at 4 °C	Post-column derivatization with ninhydrin reagent and detection by absorbance at 440 nm and 570 nm.	[37,38]
		Obtaining milk extracts and fermented milk.	[102]
HPLC reverse-phase	Analysis of GABA content using the HPLC gradient system with precolumn derivatization. Absorbance is detected at a specific wavelength depending on the derivatized complex obtained.	Pre-column derivatization protocol was applied.	[27,30,39,42,48,53,55,67,79,80,83,86,90,91,92,93,107,110,111,115,117,118,119,120,127,128,129,130,131]
		Post-column derivatization with diethyl ethoxy methylene malonate.	[52]
		Previously prescreening with gas release method.	[65]
		Previously prescreening with GABase enzymatic assay.	[45]
HPLC (without specific methodology declared)	No data found	Refers to derivatization protocols without details	[46,59,94,108,116,132]
		The authors used a fluorescence detector coupled to HPLC	[28]
UPLC (Ultra performance liquid chromatography)	The analytical gradient for the eluents and the injection volume were selected based on the UPLC equipment used in each study	The precolumn derivatization reagents were o-phthalaldehyde, AccQ·Fluor reagent kit, and diethylethoxymethylene malonate.	[41,47,133]
CE-TOFMS (Capillary electrophoresis time-of-flight mass spectrometry)	The metabolites were analyzed using a fused silica capillary with cationic and anionic buffer solutions. Electrospray ionization mass spectrometry (ESI-MS) was performed in positive and negative.	Reverse phase HPLC protocols with phenyl isothiocyanate (PITC) were used as a preparative protocol for the samples.	[49]
LC-MS (Liquid chromatography mass spectrometry)	An API 3000 triple quadrupole mass spectrometer equipped with a TurboIon Spray (SCIEX, Framingham, MA, USA) source was used. Two mobile phases were considered. The analysis was performed in positive ion mode in multiple reaction monitoring (MRM) using the following precursor/product ion combination: *m*/*z* 104 → 87.	The system included an HPLC apparatus equipped with two Series 200 micropumps and a Kinetex 2.6 µ HILIC 100 Å column (100 mm × 2.1 mm).	[58]
TLC (Thin layer chromatography) quantitative	The supernatant from the cultured cells was applied to glass plates coated with silica gel. Next, a solution of ninhydrin was sprayed directly onto the plates as a developing agent, and the plates were exposed to a heat source for a few minutes.	For GABA quantification the area and intensity of GABA spots on the plates were measured by Shimadzu CS-930 (Shimadzu, Kyoto, Japan) densitometer at 512 nm.	[113]
		The conversion rate of MSG to GABA on TLC plates. HPTLC (high-performance thin-layer chromatography) was applied. GABA spots were observed at 480 nm on HPTLC plates.	[57]
		The conversion rate of MSG to GABA was analyzed using ImageJ software (version 1.54). HPLC for quantification	[40]
		TLC for prescreening. HPLC for quantification	[35,60,61,77,78,81,82,97,101,105,106,112,134,135,136]
		TLC for prescreening. GABase enzymatic assay for quantification.	[54,68,100,121]
		TLC for prescreening. Colorimetric phenol-hypochlorite method (Berthelot) for quantification.	[99]
TLC qualitative	The sample was applied to a silica gel TLC plate. GABA and MSG were also applied as controls. The plate was exposed to a mobile phase composed of butanol, acetic acid, and distilled water. The retention factor (Rf) of each spot was calculated	Screening of GABA producing strains in TLC plates is a presumptive method, fast and cheap to identify GABA production.	[31,87]
TLC preparative	The GABA spots were scraped from the TLC plates and extracted with ethanolic cupric sulfate. The absorbance of the supernatant was read at 520 nm in an ultraviolet-visible spectrophotometer.	TLC for preparative sample extraction and cupric-sulfate development for quantification	[75,85,87]
GC-MS (Gas chromatography mass spectrometry)	The supernatant fractions were derivatized using the EZ:faast kit (Phenomenex, Inc., Torrance, CA, USA) according to the manufacturer’s recommendations. The derived samples were then analyzed by GC with a flame ionization detector.	Model: HP 5890A Gas Chromatograph, Hewlett Packard, Avondale, PA, USA	[44,137]
GABase enzymatic assay (spectrophotometric)	The GABase assay consisted of sodium sulfate, dithiothreitol, α-ketoglutarate, NADP+, and P.Fluorescence GABase reagents in Tris-HCl buffer. The absorbance of the mixture was read at 340 nm in a UV/visible spectrophotometer.	Single enzyme kit was used.	[24,66,71,73,96,138]
		The authors replaced sodium sulfate and dithiothreitol for potassium pyrophosphate.	[33]
		An enzymatic mixture of gamma-aminobutyrate, glutamate aminotransferase, and succinic semialdehyde dehydrogenase was used.	[29]
		Complemented with HPLC.	[69,70]
		Spectrophotometric assay (no further details).	[104]
Colorimetric GAD assay	The cells were washed with phosphate-buffered saline and homogenized with GAD solution, then incubated under microaerophilic conditions. The colorimetric reaction was observed visually, and the color was analyzed.	Prescreening method	[139]
Colorimetric phenol-hypochlorite method (Berthelot)	The supernatants were processed with sodium tetraborate, phenol solution, and sodium hypochlorite solution. Alcohol was added after a blue-green color appeared. The absorbance of the samples was read at 640 nm.	Authors adopted the colorimetric method according to the results of the comparison between the colorimetric method and the HPLC determination made by [140].	[84,141,142]

## Data Availability

No new data were created or analyzed in this study. Data sharing is not applicable to this article.

## References

[1] Komatsuzaki N., Shima J., Kawamoto S., Momose H., Kimura T. (2005). Production of γ-Aminobutyric Acid (GABA) by Lactobacillus Paracasei Isolated from Traditional Fermented Foods. Food Microbiol..

[2] Jena R., Choudhury P.K. (2024). Lactic Acid Bacteria in Fermented Dairy Foods: Gamma-Aminobutyric Acid (GABA) Production and Its Therapeutic Implications. Food Biosci..

[3] Yogeswara I.B.A., Maneerat S., Haltrich D. (2020). Glutamate Decarboxylase from Lactic Acid Bacteria—A Key Enzyme in Gaba Synthesis. Microorganisms.

[4] Wang X., Wang Y., Nan B., Cao Y., Piao C., Li X., Wang Y. (2024). Optimization of Fermentation for Gamma-Aminobutyric Acid (GABA) Production by *Lactiplantibacillus plantarum* Lp3 and the Development of Fermented Soymilk. LWT.

[5] Grewal J. (2020). Gamma-Aminobutyric Acid (GABA): A Versatile Bioactive Compound. Eur. J. Mol. Clin. Med..

[6] Rashmi D., Zanan R., John S., Khandagale K., Nadaf A. (2018). γ-Aminobutyric Acid (GABA): Biosynthesis, Role, Commercial Production, and Applications. Studies in Natural Products Chemistry.

[7] Market US Global GABA (Gamma-Aminobutyric Acid) Market By Type (Chemical Synthesis, Biological Fermentation), By Application (Pharmaceuticals, Food & Beverage, Animal Feeds, Others), By Distribution Channel (Direct Sales, Indirect Sales), By Region and Key Companies—Industry Segment Outlook, Market Assessment, Competition Scenario, Trends and Forecast 2024–2033. https://market.us/report/gaba-gamma-aminobutyric-acid-market/.

[8] Dhakal R., Bajpai V.K., Baek K.-H. (2012). Production of Gaba (γ-Aminobutyric Acid) by Microorganisms: A Review. Braz. J. Microbiol..

[9] Cui Y., Miao K., Niyaphorn S., Qu X. (2020). Production of Gamma-Aminobutyric Acid from Lactic Acid Bacteria: A Systematic Review. Int. J. Mol. Sci..

[10] Falah F., Vasiee A., Tabatabaei-Yazdi F., Moradi S., Sabahi S. (2024). Optimization of γ-Aminobutyric Acid (GABA) Production by *Lactobacillus* spp. from Agro-Food Waste. Biomass Convers. Biorefin..

[11] Lin Q. (2013). Submerged Fermentation of Lactobacillus Rhamnosus YS9 for G-Aminobutyric Acid (GABA) Production. Braz. J. Microbiol..

[12] Barrett E., Ross R.P., O’Toole P.W., Fitzgerald G.F., Stanton C. (2012). γ-Aminobutyric Acid Production by Culturable Bacteria from the Human Intestine. J. Appl. Microbiol..

[13] Yang S.Y., Lü F.X., Lu Z.X., Bie X.M., Jiao Y., Sun L.J., Yu B. (2008). Production of γ-Aminobutyric Acid by *Streptococcus salivarius* Subsp. Thermophilus Y_2_ under Submerged Fermentation. Amino Acids.

[14] Icer M.A., Sarikaya B., Kocyigit E., Atabilen B., Çelik M.N., Capasso R., Ağagündüz D., Budán F. (2024). Contributions of Gamma-Aminobutyric Acid (GABA) Produced by Lactic Acid Bacteria on Food Quality and Human Health: Current Applications and Future Prospects. Foods.

[15] Coda R., Rizzello C.G., Gobbetti M. (2010). Use of Sourdough Fermentation and Pseudo-Cereals and Leguminous Flours for the Making of a Functional Bread Enriched of γ-Aminobutyric Acid (GABA). Int. J. Food Microbiol..

[16] Kim J.Y., Lee M.Y., Ji G.E., Lee Y.S., Hwang K.T. (2009). Production of γ-Aminobutyric Acid in Black Raspberry Juice during Fermentation by *Lactobacillus brevis* GABA100. Int. J. Food Microbiol..

[17] Park K.B., Oh S.H. (2007). Production of Yogurt with Enhanced Levels of Gamma-Aminobutyric Acid and Valuable Nutrients Using Lactic Acid Bacteria and Germinated Soybean Extract. Bioresour. Technol..

[18] Li H., Qiu T., Huang G., Cao Y. (2010). Production of Gamma-Aminobutyric Acid by *Lactobacillus brevis* NCL912 Using Fed-Batch Fermentation. Microb. Cell Factories.

[19] Torino M.I., Limón R.I., Martínez-Villaluenga C., Mäkinen S., Pihlanto A., Vidal-Valverde C., Frias J. (2013). Antioxidant and Antihypertensive Properties of Liquid and Solid State Fermented Lentils. Food Chem..

[20] Guleria A., Chakma S. (2023). A Bibliometric and Visual Analysis of Contaminant Transport Modeling in the Groundwater System: Current Trends, Hotspots, and Future Directions. Environ. Sci. Pollut. Res..

[21] Grewal J., Khare S.K. (2017). 2-Pyrrolidone Synthesis from Γ-Aminobutyric Acid Produced by *Lactobacillus brevis* under Solid-State Fermentation Utilizing Toxic Deoiled Cottonseed Cake. Bioprocess Biosyst. Eng..

[22] Redruello B., Saidi Y., Sampedro L., Ladero V., Del Rio B., Alvarez M.A. (2021). Gaba-Producing Lactococcus Lactis Strains Isolated from Camel’s Milk as Starters for the Production of Gaba-Enriched Cheese. Foods.

[23] Villegas J.M., Brown L., Savoy de Giori G., Hebert E.M. (2016). Optimization of Batch Culture Conditions for GABA Production by *Lactobacillus brevis* CRL 1942, Isolated from Quinoa Sourdough. LWT—Food Sci. Technol..

[24] Kittibunchakul S., Yuthaworawit N., Whanmek K., Suttisansanee U., Santivarangkna C. (2021). Health Beneficial Properties of a Novel Plant-Based Probiotic Drink Produced by Fermentation of Brown Rice Milk with GABA-Producing Lactobacillus Pentosus Isolated from Thai Pickled Weed. J. Funct. Foods.

[25] Bown A.W., Shelp B.J. (1997). The Metabolism and Functions of [Gamma]-Aminobutyric Acid. Plant Physiol..

[26] Aoki H., Uda I., Tagami K., Furuya Y., Endo Y., Fujimoto K. (2003). The Production of a New Tempeh-like Fermented Soybean Containing a High Level of γ-Aminobutyric Acid by Anaerobic Incubation with Rhizopus. Biosci. Biotechnol. Biochem..

[27] Ab Kadir S., Wan-Mohtar W.A.A.Q.I., Mohammad R., Abdul Halim Lim S., Sabo Mohammed A., Saari N. (2016). Evaluation of Commercial Soy Sauce *Koji* Strains of *Aspergillus Oryzae* for γ-Aminobutyric Acid (GABA) Production. J. Ind. Microbiol. Biotechnol..

[28] Zhang Q., Sun Q., Tan X., Zhang S., Zeng L., Tang J., Xiang W. (2020). Characterization of γ-Aminobutyric Acid (GABA)-Producing Saccharomyces Cerevisiae and Coculture with *Lactobacillus plantarum* for Mulberry Beverage Brewing. J. Biosci. Bioeng..

[29] Hurtado-Romero A., Del Toro-Barbosa M., Gradilla-Hernández M.S., Garcia-Amezquita L.E., García-Cayuela T. (2021). Probiotic Properties, Prebiotic Fermentability, and GABA-Producing Capacity of Microorganisms Isolated from Mexican Milk Kefir Grains: A Clustering Evaluation for Functional Dairy Food Applications. Foods.

[30] Li S., Zhang Y., Li X., Yin P., Wang T., Li Y., Zhang K., Sheng H., Lu S., Ji H. (2022). The Effect of the Ratio of Gamma Aminobutyric Acid-Producing Saccharomyces Cerevisiae DL6–20 and Kluyveromyces Marxianus B13–5 Addition on Cheese Quality. Front. Microbiol..

[31] Moghimani M., Onyeaka H., Hashemi M., Afshari A. (2024). Evaluation of the Probiotic, Technological, Safety Attributes, and GABA-Producing Capacity of Microorganisms Isolated from Iranian Milk Kefir Beverages. Front. Microbiol..

[32] Guo X., Aoki H., Hagiwara T., Masuda K., Watabe S. (2009). Identification of High γ-Aminobutyric Acid Producing Marine Yeast Strains by Physiological and Biochemical Characteristics and Gene Sequence Analyses. Biosci. Biotechnol. Biochem..

[33] Han S.-M., Lee J.-S. (2017). Production and Its Anti-Hyperglycemic Effects of γ-Aminobutyric Acid from the Wild Yeast Strain *Pichia Silvicola* UL6-1 and *Sporobolomyces Carnicolor* 402-JB-1. Mycobiology.

[34] Sun X., Wang J., Li C., Zheng M., Zhang Q., Xiang W., Tang J. (2022). The Use of γ-Aminobutyric Acid-Producing Saccharomyces Cerevisiae SC125 for Functional Fermented Beverage Production from Apple Juice. Foods.

[35] Dikshit R., Tallapragada P. (2015). Screening and Optimization of γ-Aminobutyric Acid Production from *Monascus Sanguineus* under Solid-State Fermentation. Front. Life Sci..

[36] Tan X., Zhang Q., Liu J., Shang Y., Min Y., Sun X., Tang J. (2024). Enhanced γ-Aminobutyric Acid Production by Co-Culture Fermentation with Enterococcus Faecium AB157 and Saccharomyces Cerevisiae SC125. LWT.

[37] Siragusa S., De Angelis M., Di Cagno R., Rizzello C.G., Coda R., Gobbetti M. (2007). Synthesis of γ-Aminobutyric Acid by Lactic Acid Bacteria Isolated from a Variety of Italian Cheeses. Appl. Environ. Microbiol..

[38] Di Cagno R., Mazzacane F., Rizzello C.G., De Angelis M., Giuliani G., Meloni M., De Servi B., Gobbetti M. (2010). Synthesis of γ-Aminobutyric Acid (GABA) by *Lactobacillus plantarum* DSM19463: Functional Grape Must Beverage and Dermatological Applications. Appl. Microbiol. Biotechnol..

[39] Coelho M.C., Ribeiro S.C., Malcata F.X., Silva C.C.G. (2025). High Gamma-Aminobutyric Acid (GABA) Producing Enterococcus Malodoratus Isolated from Protected Denomination of Origin (PDO) Cheese. Int. Dairy J..

[40] Sokovic Bajic S., Djokic J., Dinic M., Veljovic K., Golic N., Mihajlovic S., Tolinacki M. (2019). GABA-Producing Natural Dairy Isolate from Artisanal Zlatar Cheese Attenuates Gut Inflammation and Strengthens Gut Epithelial Barrier in Vitro. Front. Microbiol..

[41] Franciosi E., Carafa I., Nardin T., Schiavon S., Poznanski E., Cavazza A., Larcher R., Tuohy K.M. (2015). Biodiversity and *γ*-Aminobutyric Acid Production by Lactic Acid Bacteria Isolated from Traditional Alpine Raw Cow’s Milk Cheeses. Biomed. Res. Int..

[42] Mancini A., Carafa I., Franciosi E., Nardin T., Bottari B., Larcher R., Tuohy K.M. (2019). In Vitro Probiotic Characterization of High GABA Producing Strain *Lactobacilluas brevis* DSM 32386 Isolated from Traditional “Wild” Alpine Cheese. Ann. Microbiol..

[43] Ribeiro S.C., Domingos-Lopes M.F.P., Stanton C., Ross R.P., Silva C.C. (2018). Production of -aminobutyric Acid (GABA) by *Lactobacillus otakiensis* and Other *Lactobacillus* sp. Isolated from Traditional Pico Cheese. Int. J. Dairy Technol..

[44] Lacroix N., St-Gelais D., Champagne C.P., Vuillemard J.C. (2013). Gamma-Aminobutyric Acid-Producing Abilities of Lactococcal Strains Isolated from Old-Style Cheese Starters. Dairy Sci. Technol..

[45] Santos-Espinosa A., Beltrán-Barrientos L.M., Reyes-Díaz R., Mazorra-Manzano M.Á., Hernández-Mendoza A., González-Aguilar G.A., Sáyago-Ayerdi S.G., Vallejo-Cordoba B., González-Córdova A.F. (2020). Gamma-Aminobutyric Acid (GABA) Production in Milk Fermented by Specific Wild Lactic Acid Bacteria Strains Isolated from Artisanal Mexican Cheeses. Ann. Microbiol..

[46] Huang J., Mei L., Xia J. (2007). Application of Artificial Neural Network Coupling Particle Swarm Optimization Algorithm to Biocatalytic Production of GABA. Biotechnol. Bioeng..

[47] Shan Y., Man C.X., Han X., Li L., Guo Y., Deng Y., Li T., Zhang L.W., Jiang Y.J. (2015). Evaluation of Improved γ-Aminobutyric Acid Production in Yogurt Using *Lactobacillus plantarum* NDC75017. J. Dairy Sci..

[48] Wu Q., Law Y.-S., Shah N.P. (2015). Dairy Streptococcus Thermophilus Improves Cell Viability of *Lactobacillus brevis* NPS-QW-145 and Its γ-Aminobutyric Acid Biosynthesis Ability in Milk. Sci. Rep..

[49] Hagi T., Kobayashi M., Nomura M. (2016). Metabolome Analysis of Milk Fermented by γ-Aminobutyric Acid–Producing Lactococcus Lactis. J. Dairy Sci..

[50] Chintakovid N., Singkhamanan K., Yaikhan T., Nokchan N., Wonglapsuwan M., Jitpakdee J., Kantachote D., Surachat K. (2024). Probiogenomic Analysis of *Lactiplantibacillus plantarum* SPS109: A Potential GABA-Producing and Cholesterol-Lowering Probiotic Strain. Heliyon.

[51] Nursini N.W., Antara N.S., Sugitha I.M., Sujaya I.N. (2022). Screening of Lactobacillus Rhamnosus-Producing Gamma Aminobutyric Acid (GABA) Isolated from Sumbawa Mare Milk and Its Potential Application to Increase GABA Content in Fermented Milk. Food Res..

[52] Tamés H., Sabater C., Margolles A., Ruiz L., Ruas-Madiedo P. (2023). Production of GABA in Milk Fermented by Bifidobacterium Adolescentis Strains Selected on the Bases of Their Technological and Gastrointestinal Performance. Food Res. Int..

[53] Khanlari Z., Moayedi A., Ebrahimi P., Khomeiri M., Sadeghi A. (2021). Enhancement of Γ-aminobutyric Acid (GABA) Content in Fermented Milk by Using *Enterococcus Faecium* and *Weissella Confusa* Isolated from Sourdough. J. Food Process. Preserv..

[54] Edalatian Dovom M.R., Habibi Najafi M.B., Rahnama Vosough P., Norouzi N., Ebadi Nezhad S.J., Mayo B. (2023). Screening of Lactic Acid Bacteria Strains Isolated from Iranian Traditional Dairy Products for GABA Production and Optimization by Response Surface Methodology. Sci. Rep..

[55] Hu T., Cui Y., Zhang Y., Qu X., Zhao C. (2020). Genome Analysis and Physiological Characterization of Four Streptococcus Thermophilus Strains Isolated from Chinese Traditional Fermented Milk. Front. Microbiol..

[56] Hou B., Wang H., Yan T., Shan Y., Zhou W., Zhang L., Man C., Deng Y., Jiang Y. (2016). Production for High-Vitality Starter Culture of *Lactobacillus plantarum* NDC 75017 by High Cell-Density Cultivation and Low-Temperature Vacuum Drying. Food Sci. Technol. Res..

[57] BS S., Thankappan B., Mahendran R., Muthusamy G., Femil Selta D.R., Angayarkanni J. (2021). Evaluation of GABA Production and Probiotic Activities of Enterococcus Faecium BS5. Probiotics Antimicrob. Proteins.

[58] Youssef H.A.I., Vitaglione P., Ferracane R., Abuqwider J., Mauriello G. (2023). Evaluation of GABA Production by Alginate-Microencapsulated Fresh and Freeze-Dried Bacteria Enriched with Monosodium Glutamate during Storage in Chocolate Milk. Microorganisms.

[59] Ozer M., Ozturk B., Hayaloglu A.A., Tellioglu Harsa S. (2022). Development of a Functional Chocolate Using Gamma-Amino Butyric Acid Producer Lacticaseibacillus Rhamnosus NRRL B-442. Food Biosci..

[60] Zhang Q., Zeng L., Tan X., Tang J., Xiang W. (2017). An Efficient γ-Aminobutyric Acid (GABA) Producing and Nitrite Reducing Ability of *Lactobacillus plantarum* BC114 Isolated from Chinese Paocai. Food Sci. Technol. Res..

[61] Ding J., Ba W., You S., Qi W., Su R. (2023). Development of an Oil-Sealed Anaerobic Fermentation Process for High Production of γ-Aminobutyric Acid with *Lactobacillus brevis* Isolated by Directional Colorimetric Screening. Biochem. Eng. J..

[62] Lee B.-J., Kim J.-S., Kang Y.M., Lim J.-H., Kim Y.-M., Lee M.-S., Jeong M.-H., Ahn C.-B., Je J.-Y. (2010). Antioxidant Activity and γ-Aminobutyric Acid (GABA) Content in Sea Tangle Fermented by *Lactobacillus brevis* BJ20 Isolated from Traditional Fermented Foods. Food Chem..

[63] Lu X., Chen Z., Gu Z., Han Y. (2008). Isolation of γ-Aminobutyric Acid-Producing Bacteria and Optimization of Fermentative Medium. Biochem. Eng. J..

[64] Seok J.-H., Park K.-B., Kim Y.-H., Bae M.-O., Lee M.-K., Oh S.-H. (2008). Production and Characterization of Kimchi with Enhanced Levels of γ-Aminobutyric Acid. Food Sci. Biotechnol..

[65] Wu Q., Shah N.P. (2015). Gas Release-Based Prescreening Combined with Reversed-Phase HPLC Quantitation for Efficient Selection of High-γ-Aminobutyric Acid (GABA)-Producing Lactic Acid Bacteria. J. Dairy Sci..

[66] Seo M.-J., Nam Y.-D., Park S.-L., Lee S.-Y., Yi S.-H., Lim S.-I. (2013). γ-Aminobutyric Acid Production in Skim Milk Co-Fermented with *Lactobacillus brevis* 877G and Lactobacillus Sakei 795. Food Sci. Biotechnol..

[67] Wang D., Wang Y., Lan H., Wang K., Zhao L., Hu Z. (2021). Enhanced Production of γ-Aminobutyric Acid in Litchi Juice Fermented by *Lactobacillus plantarum* HU-C2W. Food Biosci..

[68] Lim H.S., Cha I.-T., Lee H., Seo M.-J. (2016). Optimization of Gamma-Aminobutyric Acid Production by Enterococcus Faecium JK29 Isolated from a Traditional Fermented Foods. Microbiol. Biotechnol. Lett..

[69] Kim J., Yoon Y.-W., Kim M.-S., Lee M.-H., Kim G.-A., Bae K., Yoon S.-S. (2022). Gamma-Aminobutyric Acid Fermentation in MRS-Based Medium by the Fructophilic *Lactiplantibacillus plantarum* Y7. Food Sci. Biotechnol..

[70] Kim J., Lee M.-H., Kim M.-S., Kim G.-H., Yoon S.-S. (2022). Probiotic Properties and Optimization of Gamma-Aminobutyric Acid Production by *Lactiplantibacillus plantarum* FBT215. J. Microbiol. Biotechnol..

[71] Park S.-Y., Kim K.-S., Lee M.-K., Lim S.-D. (2013). Physiological Characteristics and GABA Production of *Lactobacillus plantarum* K255 Isolated from Kimchi. Korean J. Food Sci. Anim. Resour..

[72] Liu W., Li H., Liu L., Ko K., Kim I. (2022). Screening of Gamma-Aminobutyric Acid-Producing Lactic Acid Bacteria and the Characteristic of Glutamate Decarboxylase from *Levilactobacillus brevis* F109-MD3 Isolated from Kimchi. J. Appl. Microbiol..

[73] Ahn J., Park J.-Y. (2023). Potential of γ-Aminobutyric Acid-Producing *Leuconostoc Mesenteroides* Strains Isolated from Kimchi as a Starter for High-γ-Aminobutyric Acid Kimchi Fermentation. Prev. Nutr. Food Sci..

[74] Karimian E., Moayedi A., Khomeiri M., Aalami M., Mahoonak A.S. (2020). Application of High-GABA Producing *Lactobacillus plantarum* Isolated from Traditional Cabbage Pickle in the Production of Functional Fermented Whey-Based Formulate. J. Food Meas. Charact..

[75] Chen W., Xu W., Zheng X. (2015). A *Lactobacillus plantarum* Strain Newly Isolated from Chinese Sauerkraut with High γ-Aminobutyric Acid Productivity and Its Culture Conditions Optimization. Ecology.

[76] Raethong N., Santivarangkna C., Visessanguan W., Santiyanont P., Mhuantong W., Chokesajjawatee N. (2022). Whole-Genome Sequence Analysis for Evaluating the Safety and Probiotic Potential of *Lactiplantibacillus pentosus* 9D3, a Gamma-Aminobutyric Acid (GABA)-Producing Strain Isolated from Thai Pickled Weed. Front. Microbiol..

[77] Phuengjayaem S., Booncharoen A., Tanasupawat S. (2021). Characterization and Comparative Genomic Analysis of Gamma-Aminobutyric Acid (GABA)-Producing Lactic Acid Bacteria from Thai Fermented Foods. Biotechnol. Lett..

[78] Agung Yogeswara I., Kusumawati I.G.A., Sumadewi U., Rahayu E., Indrati R. (2018). Isolation and Identification of Lactic Acid Bacteria from Indonesian Fermented Foods as γ-Aminobutyric Acid-Producing Bacteria. Int. Food Res. J..

[79] Wan-Mohtar W.A.A.Q.I., Sohedein M.N.A., Ibrahim M.F., Ab Kadir S., Suan O.P., Weng Loen A.W., Sassi S., Ilham Z. (2020). Isolation, Identification, and Optimization of γ-Aminobutyric Acid (GABA)-Producing Bacillus Cereus Strain KBC from a Commercial Soy Sauce Moromi in Submerged-Liquid Fermentation. Processes.

[80] Song H.Y., Yu R.C. (2018). Optimization of Culture Conditions for Gamma-Aminobutyric Acid Production in Fermented Adzuki Bean Milk. J. Food Drug Anal..

[81] Ratanaburee A., Kantachote D., Charernjiratrakul W., Penjamras P., Chaiyasut C. (2011). Enhancement of γ-Aminobutyric Acid in a Fermented Red Seaweed Beverage by Starter Culture *Lactobacillus plantarum* DW12. Electron. J. Biotechnol..

[82] Li W., Wei M., Wu J., Rui X., Dong M. (2016). Novel Fermented Chickpea Milk with Enhanced Level of *γ*-Aminobutyric Acid and Neuroprotective Effect on PC12 Cells. PeerJ.

[83] Jin Y., Wu J., Hu D., Li J., Zhu W., Yuan L., Chen X., Yao J. (2023). Gamma-Aminobutyric Acid-Producing *Levilactobacillus brevis* Strains as Probiotics in Litchi Juice Fermentation. Foods.

[84] Xia Y., Zha M., Feng C., Li Y., Chen Y., Shuang Q. (2023). Effect of a Co-Fermentation System with High-GABA-Yielding Strains on Soymilk Properties: Microbiological, Physicochemical, and Aromatic Characterisations. Food Chem..

[85] Buatong A., Meidong R., Trongpanich Y., Tongpim S. (2022). Production of Plant-Based Fermented Beverages Possessing Functional Ingredients Antioxidant, γ-Aminobutyric Acid and Antimicrobials Using a Probiotic *Lactiplantibacillus plantarum* Strain L42g as an Efficient Starter Culture. J. Biosci. Bioeng..

[86] Karabulut G., Nemzer B.V., Feng H. (2024). γ-Aminobutyric Acid (GABA)-Enriched Hemp Milk by Solid-State Co-Fermentation and Germination Bioprocesses. Plant Foods Hum. Nutr..

[87] Viet L.Q., Pantuprakit C., Chau L.M., Trang N.P.T., Thanh N.N., Giang T.T., Riddech N., Siripornadulsil W., Phong H.X. (2025). Isolation and Selection of γ-Aminobutyric Acid Producing Lactic Acid Bacteria and Application in GABA-Enriched Tomato Juice Fermentation. Ciênc. Rural.

[88] Tamura T., Noda M., Ozaki M., Maruyama M., Matoba Y., Kumagai T., Sugiyama M. (2010). Establishment of an Efficient Fermentation System of Gamma-Aminobutyric Acid by a Lactic Acid Bacterium, *Enterococcus avium* G-15, Isolated from Carrot Leaves. Biol. Pharm. Bull..

[89] Lorusso A., Coda R., Montemurro M., Rizzello C. (2018). Use of Selected Lactic Acid Bacteria and Quinoa Flour for Manufacturing Novel Yogurt-Like Beverages. Foods.

[90] Demirbaş F., İspirli H., Kurnaz A.A., Yilmaz M.T., Dertli E. (2017). Antimicrobial and Functional Properties of Lactic Acid Bacteria Isolated from Sourdoughs. LWT—Food Sci. Technol..

[91] Venturi M., Galli V., Pini N., Guerrini S., Granchi L. (2019). Use of Selected Lactobacilli to Increase γ-Aminobutyric Acid (GABA) Content in Sourdough Bread Enriched with Amaranth Flour. Foods.

[92] Li Y., Chen X., Shu G., Ma W. (2020). Screening of Gamma-Aminobutyric Acid-Producing Lactic Acid Bacteria and Its Application in Monascus-Fermented Rice Production [Pdf]. Acta Sci. Pol. Technol. Aliment..

[93] Jin Y.H., Hong J.H., Lee J.-H., Yoon H., Pawluk A.M., Yun S.J., Mah J.-H. (2021). Lactic Acid Fermented Green Tea with *Levilactobacillus brevis* Capable of Producing γ-Aminobutyric Acid. Fermentation.

[94] Rodríguez-Sánchez S., Ramos I.M., Seseña S., Poveda J.M., Palop M.L. (2021). Potential of Lactobacillus Strains for Health-Promotion and Flavouring of Fermented Dairy Foods. LWT.

[95] Devi P.B., Rajapuram D.R., Jayamanohar J., Verma M., Kavitake D., Meenachi Avany B.A., Rani P.U., Ravi R., Shetty P.H. (2023). Gamma-Aminobutyric Acid (GABA) Production by Potential Probiotic Strains of Indigenous Fermented Foods Origin and RSM Based Production Optimization. LWT.

[96] Sa H.D., Park J.Y., Jeong S.-J., Lee K.W., Kim J.H. (2015). Characterization of Glutamate Decarboxylase (GAD) from Lactobacillus Sakei A156 Isolated from Jeot-Gal. J. Microbiol. Biotechnol..

[97] Anussara R., Kantachote D., Charernjiratrakul W., Sukhoom A. (2013). Selection of Γ-aminobutyric Acid-producing Lactic Acid Bacteria and Their Potential as Probiotics for Use as Starter Cultures in THai Fermented Sausages (*Nham*). Int. J. Food Sci. Technol..

[98] Thwe S.M., Kobayashi T., Luan T., Shirai T., Onodera M., Hamada-Sato N., Imada C. (2011). Isolation, Characterization, and Utilization of γ-Aminobutyric Acid (GABA)-Producing Lactic Acid Bacteria from Myanmar Fishery Products Fermented with Boiled Rice. Fish. Sci..

[99] TANAMOOL V., HONGSACHART P., SOEMPHOL W. (2020). Screening and Characterisation of Gamma-Aminobutyric Acid (GABA) Producing Lactic Acid Bacteria Isolated from Thai Fermented Fish (Plaa-Som) in Nong Khai and Its Application in Thai Fermented Vegetables (Som-Pak). Food Sci. Technol..

[100] Ly D., Mayrhofer S., Agung Yogeswara I., Nguyen T.-H., Domig K. (2019). Identification, Classification and Screening for γ-Amino-Butyric Acid Production in Lactic Acid Bacteria from Cambodian Fermented Foods. Biomolecules.

[101] Vo T.T.-T., Park J.-H. (2019). Characteristics of Potential Gamma-Aminobutyric Acid-Producing Bacteria Isolated from Korean and Vietnamese Fermented Fish Products. J. Microbiol. Biotechnol..

[102] Woraratphoka J., Innok S., Soisungnoen P., Tanamool V., Soemphol W. (2022). γ-Aminobutyric Acid Production and Antioxidant Activities in Fresh Cheese by *Lactobacillus plantarum* L10-11. Food Sci. Technol..

[103] Won Y.G., Yu H.-H., Chang Y.-H., Hwang H.-J. (2015). Lactic Acid Bacterial Starter Culture with Antioxidant and γ-Aminobutyric Acid Biosynthetic Activities Isolated from Flatfish-Sikhae Fermentation. J. Med. Food.

[104] Setya Prima H., Rusfidra R., Yansen F., Satrianto A. (2023). Characterization of Gamma Aminobutyric Acid-Producing Lactic Acid Bacteria Isolated from Budu Fish in Padang Pariaman West Sumatra Indonesia and Their Potentials as Probiotics. Appl. Food Biotechnol..

[105] Wu C.-H., Hsueh Y.-H., Kuo J.-M., Liu S.-J. (2018). Characterization of a Potential Probiotic *Lactobacillus brevis* RK03 and Efficient Production of γ-Aminobutyric Acid in Batch Fermentation. Int. J. Mol. Sci..

[106] Sakkaa S.E., Zaghloul E.H., Ghanem K.M. (2022). Psychobiotic Potential of Gamma-Aminobutyric Acid–Producing Marine Enterococcus Faecium SH9 from Marine Shrimp. Probiotics Antimicrob. Proteins.

[107] Ratanaburee A., Kantachote D., Charernjiratrakul W., Sukhoom A. (2013). Enhancement of γ-Aminobutyric Acid (GABA) in Nham (Thai Fermented Pork Sausage) Using Starter Cultures of Lactobacillus Namurensis NH2 and *Pediococcus pentosaceus* HN8. Int. J. Food Microbiol..

[108] Liu J., Meng F., Du Y., Nelson E., Zhao G., Zhu H., Caiyin Q., Zhang Z., Qiao J. (2020). Co-Production of Nisin and γ-Aminobutyric Acid by Engineered Lactococcus Lactis for Potential Application in Food Preservation. Front. Microbiol..

[109] Xuan Phong H., Quoc Viet L., Minh Chau L., Dang Long B.H., Thanh N.N., Tan Phat D., Truong L.D. (2023). Isolation and Selection of Lactic Acid Bacteria with the Capacity of Producing γ-Aminobutyric Acid (GABA) and Antimicrobial Activity: Its Application in Fermented Meat Product. Curr. Nutr. Food Sci..

[110] Zhang Y., Zhu M., Lu W., Zhang C., Chen D., Shah N.P., Xiao C. (2023). Optimizing *Levilactobacillus brevis* NPS-QW 145 Fermentation for Gamma-Aminobutyric Acid (GABA) Production in Soybean Sprout Yogurt-like Product. Foods.

[111] Tajabadi N., Ebrahimpour A., Baradaran A., Rahim R., Mahyudin N., Manap M., Bakar F., Saari N. (2015). Optimization of γ-Aminobutyric Acid Production by *Lactobacillus plantarum* Taj-Apis362 from Honeybees. Molecules.

[112] Jo M.-H., Hong S.-J., Lee H.-N., Ju J.-H., Park B.-R., Lee J., Kim S.-A., Eun J.-B., Wee Y.-J., Kim Y.-M. (2019). Gamma-Aminobutyric Acid Production from a Novel *Enterococcus avium* JS-N6B4 Strain Isolated from Edible Insects. J. Microbiol. Biotechnol..

[113] Yunes R.A., Poluektova E.U., Dyachkova M.S., Klimina K.M., Kovtun A.S., Averina O.V., Orlova V.S., Danilenko V.N. (2016). GABA Production and Structure of GadB/GadC Genes in Lactobacillus and Bifidobacterium Strains from Human Microbiota. Anaerobe.

[114] Li T., Luo B., Zou X., Yin J., Lv S., Wang J., Gong L. (2024). *Enterococcus avium* from a Traditional Chinese Liquor Fermentation System with the Potential for the de Novo Synthesis of GABA. LWT.

[115] Wei L., Zhao J., Wang Y., Gao J., Du M., Zhang Y., Xu N., Du H., Ju J., Liu Q. (2022). Engineering of *Corynebacterium glutamicum* for High-Level γ-Aminobutyric Acid Production from Glycerol by Dynamic Metabolic Control. Metab. Eng..

[116] Ham S., Bhatia S.K., Gurav R., Choi Y.-K., Jeon J.-M., Yoon J.-J., Choi K.-Y., Ahn J., Kim H.T., Yang Y.-H. (2022). Gamma Aminobutyric Acid (GABA) Production in Escherichia Coli with Pyridoxal Kinase (PdxY) Based Regeneration System. Enzym. Microb. Technol..

[117] Jorge J.M.P., Leggewie C., Wendisch V.F. (2016). A New Metabolic Route for the Production of Gamma-Aminobutyric Acid by *Corynebacterium glutamicum* from Glucose. Amino Acids.

[118] Zhao Z., Ding J.-Y., Ma W., Zhou N.-Y., Liu S.-J. (2012). Identification and Characterization of γ-Aminobutyric Acid Uptake System GabP *Cg* (NCgl0464) in *Corynebacterium glutamicum*. Appl. Environ. Microbiol..

[119] Baritugo K.-A., Kim H.T., David Y., Khang T.U., Hyun S.M., Kang K.H., Yu J.H., Choi J.H., Song J.J., Joo J.C. (2018). Enhanced Production of Gamma-Aminobutyrate (GABA) in Recombinant *Corynebacterium glutamicum* Strains from Empty Fruit Bunch Biosugar Solution. Microb. Cell Factories.

[120] Fan E., Huang J., Hu S., Mei L., Yu K. (2012). Cloning, Sequencing and Expression of a Glutamate Decarboxylase Gene from the GABA-Producing Strain *Lactobacillus brevis* CGMCC 1306. Ann. Microbiol..

[121] Yogeswara I.B.A., Kittibunchakul S., Rahayu E.S., Domig K.J., Haltrich D., Nguyen T.H. (2020). Microbial Production and Enzymatic Biosynthesis of γ-Aminobutyric Acid (GABA) Using *Lactobacillus plantarum* FNCC 260 Isolated from Indonesian Fermented Foods. Processes.

[122] Mo W., Cai Y., Huang S., Xiao L., Ye Y., Yang B., Zhang C., Huang Z. (2025). Enhancing Monacolin K and GABA Biosynthesis in Monascus Pilosus via GAD Overexpression: Multi-Omics Elucidation of Regulatory Mechanisms. J. Fungi.

[123] Li W., Shang J., Bao D., Wan J., Zhou C., Feng Z., Li H., Shao Y., Wu Y. (2024). Whole-Genome Sequence Analysis of *Flammulina filiformis* and Functional Validation of Gad, a Key Gene for γ-Aminobutyric Acid Synthesis. J. Fungi.

[124] Nikmaram N., Dar B., Roohinejad S., Koubaa M., Barba F.J., Greiner R., Johnson S.K. (2017). Recent Advances in *γ*-aminobutyric Acid (GABA) Properties in Pulses: An Overview. J. Sci. Food Agric..

[125] Watanabe M., Maemura K., Kanbara K., Tamayama T., Hayasaki H. (2002). GABA and GABA Receptors in the Central Nervous System and Other Organs. Int. Rev. Cytol..

[126] Kim M.-J., Kim K.-S. (2012). Isolation and Identification of γ-Aminobutyric Acid (GABA)-Producing Lactic Acid Bacteria from Kimchi. J. Korean Soc. Appl. Biol. Chem..

[127] Galli V., Venturi M., Mari E., Guerrini S., Granchi L. (2022). Gamma-Aminobutyric Acid (GABA) Production in Fermented Milk by Lactic Acid Bacteria Isolated from Spontaneous Raw Milk Fermentation. Int. Dairy J..

[128] Thuy D.T.B., Nguyen A.T., Khoo K.S., Chew K.W., Cnockaert M., Vandamme P., Ho Y.-C., Huy N.D., Cocoletzi H.H., Show P.L. (2021). Optimization of Culture Conditions for Gamma-Aminobutyric Acid Production by Newly Identified *Pediococcus pentosaceus* MN12 Isolated from ‘Mam Nem’, a Fermented Fish Sauce. Bioengineered.

[129] Sassi S., Ilham Z., Jamaludin N.S., Halim-Lim S.A., Shin Yee C., Weng Loen A.W., Poh Suan O., Ibrahim M.F., Wan-Mohtar W.A.A.Q.I. (2022). Critical Optimized Conditions for Gamma-Aminobutyric Acid (GABA)-Producing *Tetragenococcus halophilus* Strain KBC from a Commercial Soy Sauce Moromi in Batch Fermentation. Fermentation.

[130] Pakdeeto A., Phuengjayaem S., Arayakarn T., Phitchayaphon C., Tungkajiwangkoon S., Tanasupawat S. (2022). Identification of Gamma-Aminobutyric Acid (GABA)-Producing Lactic Acid Bacteria from Plant-Based Thai Fermented Foods and Genome Analysis of *Lactobacillus brevis* GPB7-4. ScienceAsia.

[131] Zou X.-Z., Gong L.-C., Li T.-T., Lv S.-Y., Wang J. (2024). Optimization of Fermentation Conditions for the Production of γ-Aminobutyric Acid by Lactobacillus Hilgardii GZ2 from Traditional Chinese Fermented Beverage System. Bioprocess. Biosyst. Eng..

[132] Jiang D., Ji H., Ye Y., Hou J. (2011). Studies on Screening of Higher γ-Aminobutyric Acid-Producing Monascus and Optimization of Fermentative Parameters. Eur. Food Res. Technol..

[133] Valenzuela J.A., Vázquez L., Rodríguez J., Flórez A.B., Vasek O.M., Mayo B. (2024). Phenotypic, Technological, Safety, and Genomic Profiles of Gamma-Aminobutyric Acid-Producing Lactococcus Lactis and Streptococcus Thermophilus Strains Isolated from Cow’s Milk. Int. J. Mol. Sci..

[134] Sanchart C., Rattanaporn O., Haltrich D., Phukpattaranont P., Maneerat S. (2017). Lactobacillus Futsaii CS3, a New GABA-Producing Strain Isolated from Thai Fermented Shrimp (Kung-Som). Indian J. Microbiol..

[135] Yu H.-H., Choi J.H., Kang K.M., Hwang H.-J. (2017). Potential of a Lactic Acid Bacterial Starter Culture with Gamma-Aminobutyric Acid (GABA) Activity for Production of Fermented Sausage. Food Sci. Biotechnol..

[136] Lee J.-S., Kim K.-S. (2023). Optimization of Culture Conditions for and Assessment of Kimchi-Originated Lactic Acid Bacterial Isolates toward Their Extracellular GABA-Producing Ability. Emir. J. Food Agric..

[137] Mousavi R., Mottawea W., Hassan H., Gomaa A., Audet M.-C., Hammami R. (2022). Screening, Characterization and Growth of γ-Aminobutyric Acid-Producing Probiotic Candidates from Food Origin under Simulated Colonic Conditions. J. Appl. Microbiol..

[138] De Oliveira F.L., Salgaço M.K., de Oliveira M.T., Mesa V., Sartoratto A., Peregrino A.M., Ramos W.S., Sivieri K. (2023). Exploring the Potential of Lactobacillus Helveticus R0052 and Bifidobacterium Longum R0175 as Promising Psychobiotics Using SHIME. Nutrients.

[139] Jovanović M., Vojvodić P., Petrović M., Radić D., Mitić-Ćulafić D., Kostić M., Veljović S. (2022). Yogurt Fortified with GABA-Producing Strain and Ganoderma Lucidum Industrial Waste. Czech J. Food Sci..

[140] Tang C.Y., Wang T., Tu J., Liu G.H., Li P., Zhao J. (2018). Comparison of Colorimetry and HPLC for Determination of γ-Aminobutyric Acid in Mulberry Leaf Tea. Food Sci..

[141] Zhuo J., Xuan J., Chen Y., Tu J., Mu H., Wang J., Liu G. (2023). Increase of γ-Aminobutyric Acid Content and Improvement of Physicochemical Characteristics of Mulberry Leaf Powder by Fermentation with a Selected Lactic Acid Bacteria Strain. LWT.

[142] Xuan J., Han X., Che J., Zhuo J., Xu J., Lu J., Mu H., Wang J., Tu J., Liu G. (2024). Production of γ–Aminobutyric Acid–Enriched Sourdough Bread Using an Isolated *Pediococcus pentosaceus* Strain JC30. Heliyon.

[143] Zhuang K., Zhang J., Fan C., Yao Z., Zhang Z. (2023). Determination of γ-Aminobutyric Acid in Fermented Soybean Products by HPLC Coupled with Pre-Column Derivatization. J. Food Compos. Anal..

[144] Cunha D.S., Coelho M.C., Ribeiro S.C., Silva C.C.G. (2022). Application of Enterococcus Malodoratus SJC25 for the Manufacture of Whey-Based Beverage Naturally Enriched with GABA. Foods.

[145] Le Vo T.D., Kim T.W., Hong S.H. (2012). Effects of Glutamate Decarboxylase and Gamma-Aminobutyric Acid (GABA) Transporter on the Bioconversion of GABA in Engineered Escherichia Coli. Bioprocess Biosyst. Eng..

[146] Duranti S., Ruiz L., Lugli G.A., Tames H., Milani C., Mancabelli L., Mancino W., Longhi G., Carnevali L., Sgoifo A. (2020). Bifidobacterium Adolescentis as a Key Member of the Human Gut Microbiota in the Production of GABA. Sci. Rep..

[147] Farrah Wahida O.A., Nazamid Saari N.S., Fatamah Abu Bakar F.A.B., Abdulamir A.S., Abdulkarim Sabo Mohammed A.S.M., Yazid Abdul Manap Y.A.M., Anwarul Hidayah Zulkifli A.H.Z. (2009). Novel, Practical and Cheap Source for Isolating Beneficial γ-Aminobutyric Acid-Producing Leuconostoc NC5 Bacteria. Res. J. Med. Sci..

[148] Han M., Liao W., Wu S., Gong X., Bai C. (2020). Use of Streptococcus Thermophilus for the in Situ Production of γ-Aminobutyric Acid-Enriched Fermented Milk. J. Dairy Sci..

[149] Syu K.-Y., Lin C.-L., Huang H.-C., Lin J.-K. (2008). Determination of Theanine, GABA, and Other Amino Acids in Green, Oolong, Black, and Pu-Erh Teas with Dabsylation and High-Performance Liquid Chromatography. J. Agric. Food Chem..

[150] Li N., Liu Y., Zhao Y., Zheng X., Lu J., Liang Y. (2016). Simultaneous HPLC Determination of Amino Acids in Tea Infusion Coupled to Pre-Column Derivatization with 2,4-Dinitrofluorobenzene. Food Anal. Methods.

[151] Bao R., Huang L., Andrade J., Tan W., Kibbe W.A., Jiang H., Feng G. (2014). Review of Current Methods, Applications, and Data Management for the Bioinformatics Analysis of Whole Exome Sequencing. Cancer Inform..

[152] Surachat K., Deachamag P., Kantachote D., Wonglapsuwan M., Jeenkeawpiam K., Chukamnerd A. (2021). In Silico Comparative Genomics Analysis of *Lactiplantibacillus plantarum* DW12, a Potential Gamma-Aminobutyric Acid (GABA)-Producing Strain. Microbiol. Res..

[153] Zhang Q., Xu B., Min Y., Liu J., Shang Y., Lan X., Xiang W., Tang J. (2025). Evaluation of the Safety and Probiotic Properties of GABA-Producing Enterococcus Faecium AB157 Based on Whole Genome and Phenotype Analysis. LWT.

[154] Feng Y., Zhang Y., Liu C., Li Y., Miao S., Grimi N., Cao H., Guan X. (2024). Metabolism, Application in the Food Industry, and Enrichment Strategies of Gamma-Aminobutyric Acid. Trends Food Sci. Technol..

[155] Heli Z., Hongyu C., Dapeng B., Yee Shin T., Yejun Z., Xi Z., Yingying W. (2022). Recent Advances of γ-Aminobutyric Acid: Physiological and Immunity Function, Enrichment, and Metabolic Pathway. Front. Nutr..

[156] Ahmad S., Fariduddin Q. (2024). “Deciphering the Enigmatic Role of Gamma-Aminobutyric Acid (GABA) in Plants: Synthesis, Transport, Regulation, Signaling, and Biological Roles in Interaction with Growth Regulators and Abiotic Stresses.”. Plant Physiol. Biochem..

[157] Yuan D., Wu X., Gong B., Huo R., Zhao L., Li J., Lü G., Gao H. (2023). GABA Metabolism, Transport and Their Roles and Mechanisms in the Regulation of Abiotic Stress (Hypoxia, Salt, Drought) Resistance in Plants. Metabolites.

[158] Milon R.B., Hu P., Zhang X., Hu X., Ren L. (2024). Recent Advances in the Biosynthesis and Industrial Biotechnology of Gamma-Amino Butyric Acid. Bioresour. Bioprocess..

[159] Xu N., Wei L., Liu J. (2017). Biotechnological Advances and Perspectives of Gamma-Aminobutyric Acid Production. World J. Microbiol. Biotechnol..

[160] Diez-Gutiérrez L., San Vicente L., Luis L.J., Villarán M.d.C., Chávarri M. (2020). Gamma-Aminobutyric Acid and Probiotics: Multiple Health Benefits and Their Future in the Global Functional Food and Nutraceuticals Market. J. Funct. Foods.

[161] 24 Chemical Research Gamma-Aminobutyric Acid (GABA) Market, Global Outlook and Forecast 2022–2028. https://www.24chemicalresearch.com/reports/179181/global-gammaaminobutyric-acid-forecast-market-2022-2028-590.

[162] Verified Market Research Gamma-Aminobutyric Acid (GABA) Market Size and Forecast. https://www.verifiedmarketresearch.com/product/gaba-market/#:~:text=Gamma%2DAminobutyric%20Acid%20(GABA)%20Market%2C%20By%20Geography&text=On%20the%20basis%20of%20regional,followed%20by%20Canada%20and%20Mexico.

[163] Amaresan N., Dharumadurai D., Babalola O.O. (2023). Food Microbiology Based Entrepreneurship: Making Money from Microbes.

